# Bacterial and Green Algal Communities of Norwegian Birch Tree Bark

**DOI:** 10.1007/s00248-026-02841-z

**Published:** 2026-07-21

**Authors:** Jaelle C. Brealey, Marie L. Davey, Bård Pedersen

**Affiliations:** Land and Biodiversity, Norwegian Institute for Nature Research, Trondheim, Norway

**Keywords:** Bark microbiome, Bark microbiota, *Betula*, Green algae, Metabarcoding

## Abstract

Tree bark hosts a unique community of microorganisms, distinct from that of other plant tissues and from the microbial associates of macrophytic epiphytes. These corticolous communities are important components of tree-associated food webs. However, the factors influencing corticolous microbial diversity are understudied. We used amplicon sequencing to characterise and compare the bacterial and aerophytic algal communities colonising the bark of birch trees at three sites in southern Norway with differing histories of nitrogen deposition. Within each site, we investigated associations between the corticolous community and variation in placement on the trunk, including north-facing vs. south-facing aspect, height and trunk diameter. While the most abundant bacterial and algal taxa were similar across the three sites, there were clear differences among sites. Aspect had a strong effect at the most oceanic and open site, with increased abundance of cyanobacteria at northern compared to southern aspects. We observed a higher abundance of nitrogen-fixing cyanobacteria at the site where historical nitrogen deposition had been lowest and correspondingly higher abundance of non-diazotrophic green algae where historical nitrogen deposition had been highest. Generally, the bacterial and algal communities followed similar patterns and co-occurrence network analysis revealed that algal taxa were interspersed among bacterial clusters. Our study provides the first characterisation of both the bacterial and aerophytic algal bark-associated communities of birch, one of the most common and ecologically important trees in Norway. Our results add to the growing body of literature demonstrating that tree bark supports a complex microbial community that varies among sites.

**Supplementary Information:**

The online version contains supplementary material available at https://doi.org/10.1007/s00248-026-02841-z.

## Introduction

Plants provide substrate and habitat for a wide variety of microorganisms including fungi, algae, and bacteria, which live both as epiphytes on the plant’s surface, but also as endophytes within their tissues [1, 2]. The bark of woody shrubs and trees in particular provides a variety of microniches suitable for microorganisms while also hosting macrophytic epiphytes (e.g. lichens, mosses, and epiphytic vascular plants) that have their own microbial associates. These corticolous communities are important components of tree-associated food webs, with algae providing significant primary production that partly supports fungal and bacterial communities, which together are vital components of the food web for microarthropods and microscopic metazoans [3]. Furthermore, bacteria growing on the surface of trees are increasingly recognised as playing a role in global biogeochemical cycles. Corticolous microbiota can metabolise climate-active gases like methane [4], while bark-associated diazotrophic bacteria are a typically overlooked component of nitrogen input into forest ecosystems [5]. Tree bark is a unique habitat compartment among plants as it is temporally persistent on the scale of years to centuries, but is subject to harsh environmental conditions including repeated desiccation-hydration cycles, high irradiance, and periodic extreme temperatures [6]. As such, the microbial communities associated with the bark of plants are unique and well differentiated from that associated with leaves and other plant tissues [7], as well as from microbial communities inhabiting other epiphytes [8].

Although relatively few studies have addressed patterns in corticolous microbial diversity, several factors have been identified as playing a role in shaping these fungal, algal, and bacterial communities. Site dependent factors often contribute considerably to structuring corticolous community diversity across landscape level scales [9]. While the same higher-level taxonomic groups of aerophytic algae and bacteria tend to dominate corticolous microbial communities across significant geographic scales [10], there is evidence for tree host specificity for some taxa [3, 11, 12], which is likely linked to tree-specific microhabitat characteristics. For example, bark roughness contributes to microhabitat diversity and has been linked to algal colony density and community composition [13–15]. Fungal, algal, and bacterial communities have all been found to vary significantly with tree size and presumably age [16], as well as bark pH, which varies substantially between host species [14]. Microhabitat conditions on specific trees have also been linked to alpha and beta diversity of microbial communities. For example, red oak bark communities have been linked to north-facing vs. south-facing aspects on the trunk [17], while relative humidity, temperature, and light availability have been shown to impact beech bark community complexity and structure [10]. Furthermore, these communities are not stable through time, with distinct seasonal fluctuations documented in the presence and abundance of bacteria and aerophytic algae [9, 15]. Finally, the communities can be structured by complex biotic interactions. For example, significant co-exclusion has been observed between corticolous bacteria and fungi [3].

Epiphytic organisms, particularly lichens and mosses, are known to be particularly sensitive to air pollution, and can be used as bioindicators of air quality [18, 19]. This sensitivity is partly due to the specific adaptations this group needs to survive the relatively harsh conditions of the bark environment, but also to the nature of the corticolous habitat. Dry deposition of air pollutants on tree bark results in accumulation of those compounds, as they adsorb into the bark, leading to higher exposure levels [20, 21]. Deposition of nitrates, sulphates, and ammonia can change bark pH, as well as the nutrient availability in the habitat [22]. While epiphytic algal communities have not specifically been used as bioindicators of air quality, there are numerous studies documenting shifts in aerophytic algal community composition, as well as population density in response to air pollution. For example, Nováková and Neustupa [23] observed short term NO_2_ pollution from traffic increased coverage of biofilm-forming algae on conifer needles in an urban setting. Similarly, nitrogen deposition has been linked to significant increases in populations of a putatively nitrophilic green alga colonising needles and twigs of conifers in Denmark [24] and Finland [25]. Freystein et al. [26] identified algal indicator species for ozone and particulate matter pollution in urban settings, and posited that aerophytic algae could also provide a basis for a biomonitoring system for air pollution.

While bark-associated microbial communities have received increasing attention in recent years, their taxonomic composition and ecological structuring remain comparatively poorly characterised, particularly in boreal systems and for specific host tree species. Birch (*Betula pubescens* and *B. pendula*) is among the most widespread and ecologically important forest-forming tree taxa in Norway [27]. It is the most abundant tree genus nationally in terms of forest area, with *B. pubescens* dominating extensive forest regions, particularly in western and northern parts of the country [28]. However, little is known about the corticolous microbiota of Norwegian birch, as previous investigations of birch bark have focused only on macroscopic epiphytes [29]. As part of a Norwegian national monitoring program, epiphytic lichens and mosses growing on birch were monitored from 1990 to 2020 at several sites covering most parts of the country [29]. During this period, aerophytic algae were observed to expand and replace other epiphytic organisms at sites that had experienced high levels of historical nitrogen deposition [30]. This expansion progressed from north-facing to south-facing bark surfaces [31]. In contrast, aerophytic algae were not recorded at other monitored sites with considerably lower levels of historical nitrogen deposition [30]. The taxonomic composition of the aerophytic algal community was not assessed within the monitoring program. In this study, we therefore used amplicon sequencing to investigate the bacterial and aerophytic algal communities colonising the bark of birch trees from three sites in southern Norway with different histories of nitrogen deposition. At the individual tree level, we investigated if microhabitat variation related to tree size, and if aspect (northern- vs. southern-facing and eastern- vs. western-facing) or height on the tree trunks can be linked to community alpha and beta diversity. At the site level, we investigated how the balance between nitrogen-fixing cyanobacteria and non-diazotrophic green algae differs among sites with high vs. low histories of nitrogen deposition.

## Methods

### Study Sites and Plots

Based on observations from a prior epiphyte monitoring program [29, 32], three sampling sites were selected in southern Norway (Fig. S1). Climate at all three sites is currently transitioning from a cold climate to temperate climate, with warm summers and no dry season [33] (Fig. S1, Table S1). Tjeldbergodden is located on the coast, while Lund and Solhomfjell are further inland. The sites have been exposed to varying levels of long-range transported sulfur and nitrogen compounds since the industrialisation of Europe. Lund was selected as the site with the highest deposition, where increased aerophytic algae abundance has also been observed [29, 30] (Fig. S2), while Tjeldbergodden was selected as the site with the lowest deposition, with Solhomfjell as an intermediate site (Table S1). However, total deposition of both sulfur and nitrogen has gradually declined at all sites over the past 30 years, reaching significantly lower levels [34–36].

Five study plots were sampled at Lund and Solhomfjell and three plots were sampled at Tjeldbergodden (Table S2), based on observations from the prior monitoring program. Plots at Lund were located in mature birch forests and plots at Solhomfjell were in birch tree stands within pine and spruce forests. All plots from both sites were within legally protected areas. One plot at Tjeldbergodden was located in mature birch forest while two were in birch stands surrounded by coastal heathland. All three plots at Tjeldbergodden were closer to rural settlements, however without visible signs of local, anthropogenic influences on the forest stands. All plots in the present study were part of previously completed epiphyte monitoring programs [29, 32] (Fig. S2). Plot selection is described further in Supplementary Material.

### Sampling

Sampling of bark from birch trunks (*B. pubescence* and *B. pendula*) was done according to a stratified, random sampling scheme to ensure an even sampling of bark with respect to aspect (compass direction) on the trunk, and a random sample with respect to both plots and candidate birch trees within plots (details in Supplementary Material). Sampling was limited to trees with circumferences > 30 cm and heights on the trunk section 130–185 cm above ground level. Between one and seven trees were sampled per plot and between one and four samples were taken per tree. Thin strips of bark, covering approximately 4 cm x 2 cm of the surface, were obtained with a cleaned scalpel while wearing disposable gloves. The scalpel was washed with 96% ethanol before each sample was taken, and new gloves were used. Bark samples were placed directly in a coffee filter, and the filter thereafter in a paper bag and sealed. After sampling, the height above ground and aspect, using a compass, of the mid-sample point were recorded. The bark samples were collected in 2022 on May 31–June 1 at Lund, June 2–3 at Solhomfjell, and November 3 at Tjeldbergodden. All bark samples were taken under dry weather conditions. Samples were stored at -20 °C before processing in the laboratory. For sequencing, 35 bark samples from each of Lund and Solhomfjell were included, while 25 were included from Tjeldbergodden, due to challenges with sampling in the field.

### DNA Extraction, Library Build and Sequencing

DNA extractions and library construction were carried out at the Center for Biodiversity Genetics (NINAGEN) at Norwegian Institute for Nature Research. Epiphytic algal and bacterial communities from each bark strip were sampled with a moistened swab, homogenised with a FastPrep-24^™^ instrument (MP Biomedicals) and extracted using the KingFisher Tissue kit (ThermoFisher Scientific) as per the manufacturer’s instructions (further details in Supplementary Material). A negative control (buffer only) was included for homogenisation and extraction.

PCR amplification of the target genes was performed on samples, extraction negative control and PCR negative controls (water only), using the v4 region of the 16S rDNA gene for bacteria [37, 38] and the ITS2 region for green algae [16]. Primer sequences and PCR reaction details are provided in Supplementary Material. PCR products were cleaned using magnetic beads (Mag-Bind RxnPure Plus), before an indexing PCR was performed with IDT for Illumina UD index kits (Illumina, USA) following the manufacturer’s instructions. PCR products were again cleaned using magnetic beads, then amplicons were pooled in equimolar amounts and sequenced in a paired-end 250 bp run using a NovaSeq SP flow cell on an Illumina NovaSeq 6000 instrument at the Norwegian Sequencing Center, University of Oslo, Norway.

### Bioinformatics

Primer sequences were removed from the forward and reverse reads using cutadapt v3.7 [39]. Sequences were then processed in R v4.4.1 [40] using the pipeline DADA2 v1.32.0 [41], including quality trimming and filtering, error estimation, inferring of amplicon sequence variants (ASVs), merging of forward and reverse reads and removal of chimeric sequences (details in Supplementary Material).

Algal taxonomy was assigned to the ITS dataset up to the genus-level using blastn v2.14.0 [42] against the NCBI nucleotide non-redundant database (downloaded on 23 October 2023). ASVs were assigned taxonomy from the blast search based on best hits with > 75% identity and > 75% minimum coverage. ASVs were retained as green algae if they were assigned to the classes Chlorophyceae, Trebouxiophyceae, Ulvophyceae, Klebsormidiophyceae and Zygnemophyceae.

Bacterial taxonomy was assigned to the 16S dataset up to the genus-level using the RDP naïve Bayesian classifier implemented in DADA2 [43], with a minimum of 80 bootstraps and the SILVA non-redundant (99% identity) database v138.1 (DOI: https://doi.org/10.5281/zenodo.4587946, generated March 7, 2021; [44]). This database uses an older taxonomy, including outdated phyla names, such as ‘Cyanobacteria’, which has now been updated to ‘Cyanobacteriota’, and ‘Proteobacteria’, which is now ‘Pseudomonadota’ and the order Rhizobiales, which is now Hyphomicrobiales [45, 46]. However, for consistency with the version of the database that we used, we continue to use the older names throughout. ASVs that could not be assigned taxonomy at the phylum-level were removed, as were ASVs assigned to Eukaryotes or mitochondria (order Rickettsiales, family Mitochondria). ASVs which were assigned to the phylum Cyanobacteria also included chloroplast sequences. Thus, all ASVs assigned to the phylum Cyanobacteria were compared to the NCBI nucleotide non-redundant database [47] using blastn v2.14.0 [42]. ASVs were assigned taxonomy from the blast search based on best hits with > 97% identity and > 80% minimum coverage. ASVs assigned to the class-level of the phylum Cyanobacteria in both methods were retained as true cyanobacteria. ASVs assigned to chloroplasts from plants were further grouped into ‘green algae’ (blast assignments to the class Chlorophyceae, Trebouxiophyceae or Zygnemophyceae) or ‘bryophytes’ (blast assignments to the class Andreaeopsida, Bryopsida, Sphagnopsida or Jungermanniopsida). Since Bryopsida and Jungermanniopsida were by far the most abundant of the bryophytes, we assume that these reads originate from epiphytic bryophytes, while the remaining low-abundant classes are likely spores.

After processing, five bacterial ASVs were detected in the negative control and at low relative abundance in the samples. They were removed from the dataset as potential laboratory contaminants. Four algal ASVs were detected at low abundance in the negative control and at high abundance in the samples, suggesting DNA carryover during processing. These algal ASVs were retained for the analysis.

### Statistical Analysis

All statistical analysis was performed in RStudio v2022.12.1 using R v4.4.1 [40]. Covariates included site (Lund, Solholmfjell and Tjeldbergodden), plot within site (5 plots each in Lund and Solhomfjell, 3 in Tjeldbergodden), sampling height (cm) and tree size, measured as diameter at breast height (DBH, in cm; only available for Lund and Solhomfjell). Aspect (compass direction) was originally recorded in degrees, with 0°/400° representing north and 200° representing south. This cyclical variable was transformed into two continuous variables representing east–west and north–south aspects, using the sine and cosine, respectively, of 2 × π × aspect / 400° [15]. A summary of each covariate by plot within site is provided in Fig. S3. In all subsequent analyses testing for differences among sites, sine-transformed aspect, cosine-transformed aspect and height were included as covariates, with post-hoc pairwise comparisons between sites calculated with the glht function in the multcomp R package. In all analyses investigating differences within each site, sine-transformed aspect, cosine-transformed aspect, height, DBH (for Lund and Solhomfjell) and plot were included as covariates, with post-hoc pairwise comparisons between plots.

For visualisation, taxa counts were transformed to relative abundance using total sum scaling. For ordination, counts were transformed using the robust centred log-ratio (rCLR) method in the microbiome R package [48, 49]. Aitchison (Euclidean) distances were calculated and PCoAs generated with the phyloseq R package [50]. To compare beta diversity among sites or plots, PERMANOVA was performed on the Aitchison distances with the adonis2 function in the R package vegan, with by=terms and 999 permutations using tree ID as a blocking variable [51]. An interaction term between aspect and site or plot was included. Since the parameter ‘by=terms’ is sensitive to the order of variables in the model, we first used ‘by=margin’ (with no interaction term) to determine the order of variables in the final PERMANOVAs, based on the amount of variation explained by each variable (R^2^ value). The PERMANOVA was repeated for samples within each site, to investigate the effect of DBH in Lund and Solhomfjell. Ordinations and PERMANOVAs were performed on the genus- and ASV-levels. Tests of homogeneity of multivariate dispersion were performed for site and plot with the betadisper function in vegan. Alpha diversity was estimated for both datasets at the genus-level using iNEXT accumulation curves on a per-sample basis, estimating richness (q = 0) with 100 bootstraps [52]. Differences in richness among sites and within each site were investigated using linear models.

To identify bacterial and green algal taxa associated with site or microhabitat variation, differential abundance analysis was carried out using Analysis of Compositions of Microbiomes with Bias Correction 2 (ANCOM-BC2) method implemented in the ANCOMBC R package [53]. ANCOM-BC2 includes both the exclusion of structural zeros and a sensitivity analysis for the pseudo-count addition required for the log transformation of zero counts. All ANCOMBC models were run at the genus-level with the exclusion of taxa with < 10% prevalence in the dataset, taxa containing structural zeros in some groups and samples with < 1000 reads. P values were adjusted to control the false discovery rate (FDR) from multiple hypothesis testing using the Holm-Bonferroni method (“holm”). Otherwise, ANCOMBC was run with default parameters, for all sites and within each site. Taxa were classed as significantly differentially abundant if they passed the pseudo-count sensitivity analysis and had an adjusted p value < 0.05 in either the primary or pairwise test (depending on the model). A pairwise directional test was included to identify differences between each site, controlling for mixed directional FDR using the “holm” method and 100 bootstraps.

We verified the field observations of increased algal dominance at Lund by re-analysing the composition of the epiphyte community from the last year of the monitoring program at each site (Fig. S2; 2011 for Tjeldbergodden, 2016 for Lund and 2020 for Solhomfjell), using the Wilcoxon rank sum test to compare percent coverage of aerophytic algae (summarised per plot) between Lund and the other two sites. We also compared the percent coverage of bryophytes among sites. To see whether similar patterns were observed up to eleven years later in our metabarcoding data, we used the 16S dataset to investigate the relative abundance of cyanobacteria and photosynthetic eukaryotes at each site. We calculated the number of 16S reads in each sample assigned to bryophytes, green algae and cyanobacteria, divided by the total number of reads from these organisms in the 16S dataset. Differences among sites and within sites were investigated in linear models. We also used Spearman’s correlation tests to compare the earlier field observations for aerophytic algae and bryophytes (% cover) with the later 16S metabarcoding relative abundances for green algae and bryophytes (mean abundance per plot).

To infer putative interactions among taxa, we followed the methods of Dreyling et al. [16] to generate a genus-level network for each of the bacterial and algal communities, as well as a cross-domain network with both datasets. Networks were generated with R package SpiecEasi, which implements the SPIEC-EASI method for inferring sparse and compositionally robust microbial networks [54]. Parameters for the main spiec.easi algorithm included using Meinshausen-Bühlmann’s neighbourhood selection (method=“mb”) [55] and model selection using Bounded StARS (sel.criterion=“bstars) [56] on 50 subsamples (rep.num = 50), with lambda.min.ratio = 0.01, nlambda = 100 and pulsar.select=TRUE. We used the function multi.spiec.easi to calculate the cross-domain network [57]. Network properties were extracted using the R packages NetCoMi [58] and igraph [59]. Nodes were clustered into modules using the NetCoMi function netAnalyze, with the fast greedy clustering method [60]. Hub taxa were identified as those with the highest betweenness centrality (those in the 95% quantile). We compared key network properties of each observed network against a null model generated using 1000 permutations of the Erdős–Rényi random graph implemented in igraph, which preserves the size and density of the observed network while randomising node connections [61]. A one-sided p-value was calculated for each metric, calculated as the number of times the random metric was equal or above the observed metric, divided by the number of permutations. For the cross-domain network, we also calculated domain assortativity using igraph, to determine how preferentially attached bacterial nodes were to other bacterial nodes compared to algal nodes. We compared the observed domain assortativity to 1000 permutations of random node assignment to either domain in the cross-domain network.

## Results

### Bacterial Community

**Fig. 1:**
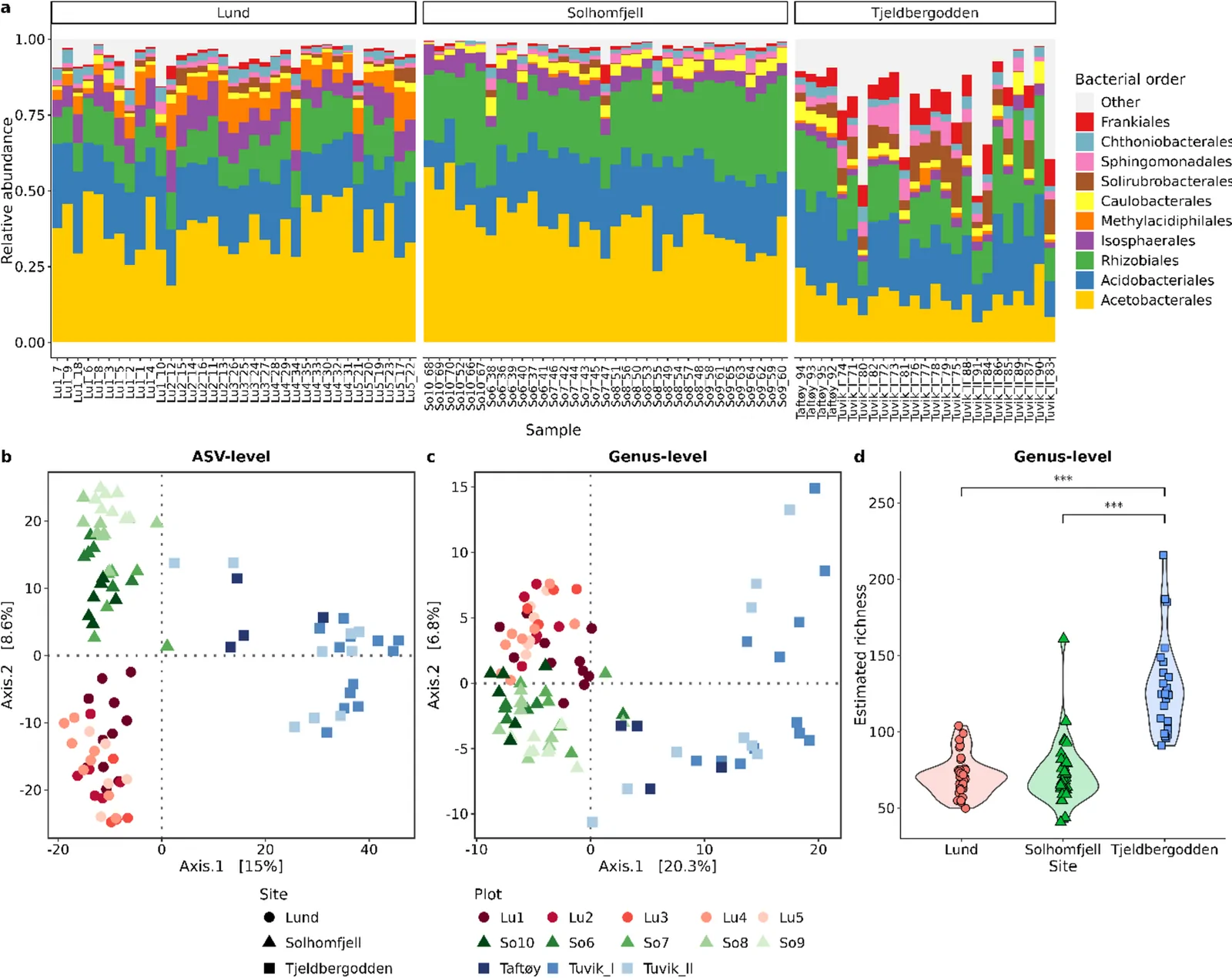
Bacteria community composition differs by site. **a**) Relative abundance of the most abundant orders in each sample. Samples are grouped by site and ordered by plot, as indicated by the sample labels. The ten most abundant orders are shown, with all remaining orders grouped into ‘other’. **b**–**c**) PCoA of the community based on ASV abundances (**b**) and genera abundances (**c**), based on Aitchison distances from robust CLR transformed abundance values. Shapes indicate site while colours indicate plot. **d**) Violin plots of genus-level richness among sites. Statistically significant differences among sites are indicated by brackets (*p* < 0.001: ***)

In the 16S dataset, 11,948 ASVs were identified and assigned to 503 bacterial genera (Data S1). The bacterial community was dominated by the phyla Proteobacteria and Acidobacteriota at all three sites. At the order-level, Acetobacteriales, Acidobacteriales and Rhizobiales were the most common taxa (Fig. 1a), while at the genus-level, 1174-901-12 (order Rhizobiales), *Granulicella* (order Acidobacteriales) and *Acidiphilium* (order Acetobacterales) were most common in all three sites (Fig. S4). Genus-level richness was significantly higher in Tjeldbergodden compared to Lund and Solhomfjell (Fig. 1d, Table S3). In the PERMANOVAs, beta diversity significantly differed among sites and plots at the ASV-level, though differences were not significant at the genus-level (Fig. 1b-c; Table 1, Table S4). However, significantly different dispersions were detected among sites, reflecting more heterogenous communities in Tjeldbergodden and more homogeneous communities in Lund (*p* < 0.002 for all comparisons). Dispersions among plots within each site were similar (*p* > 0.05). Thus, the significant PERMANOVA results indicate differences in both average site community composition at the ASV-level and differences in within-site (but not within-plot) sample variation. In the differential abundance analysis, 84 genera were associated with site (Table S5, Figs. S5 and S6).

**Fig. 2:**
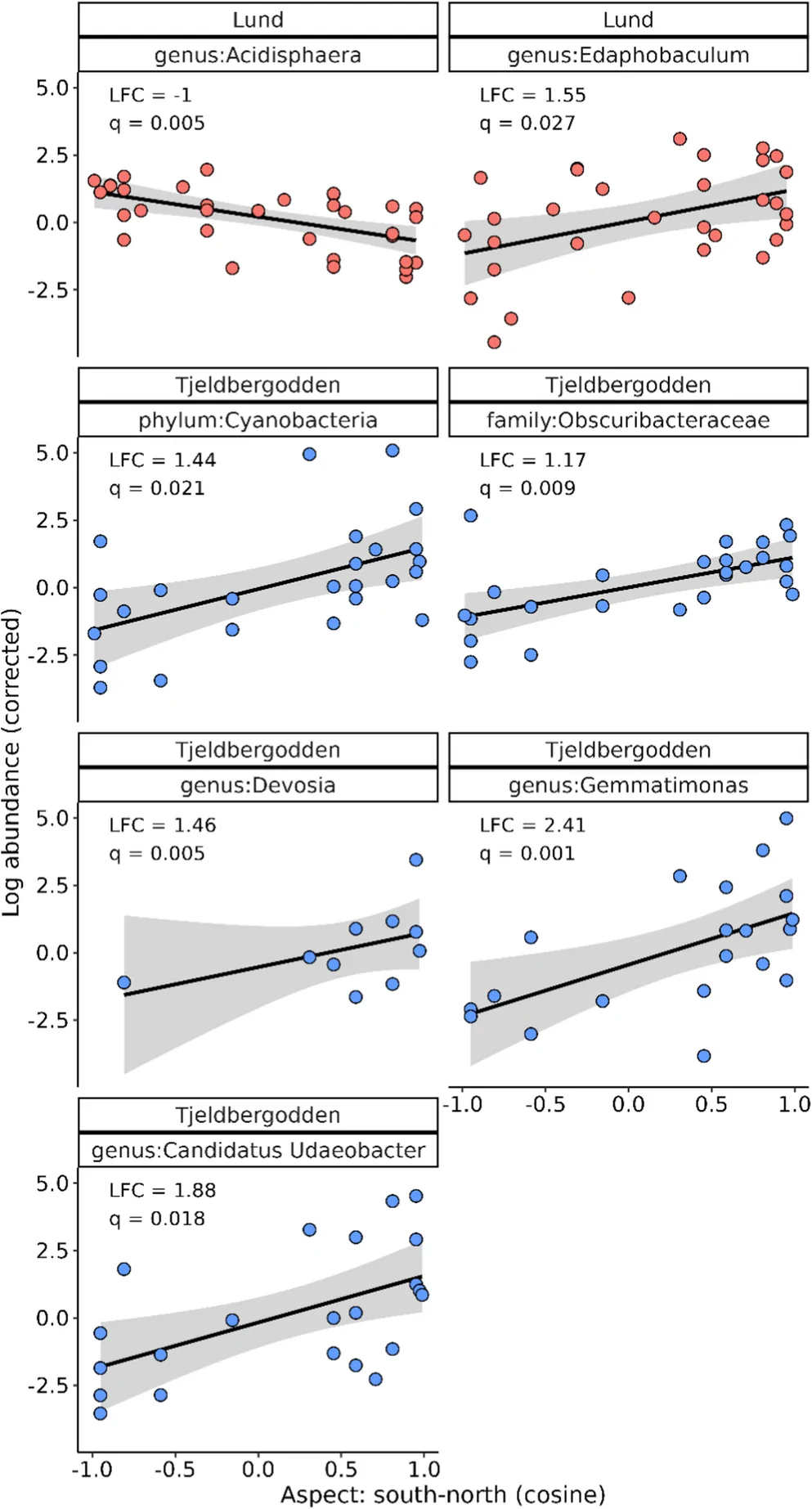
Bacterial taxa associated with south–north aspects within a site. Two genera were associated with aspect within Lund (red). Three genera together with the unclassified ASVs from the phylum Cyanobacteria and the cyanobacterial family Obscuribacteraceae were associated with aspect within Tjeldbergodden (blue). Bacterial abundance has been bias-corrected and log-transformed by ANCOM. Trend lines (black) with confidence intervals (grey shading) were generated using the lm method of the function geom_smooth within the R package ggplot2. The log-fold-change (LFC) and adjusted p value (q) for each ANCOM test is shown in the upper left corner of each plot

Genus-level richness was not associated with aspect, sampling height or DBH in any site (Table S3). Beta diversity significantly differed by south-north aspect (Table 1). This effect was site-dependent, particularly for ASV-level beta diversity, with significant interactions between site and aspect (Table 1), driven by associations in Lund (R^2^ = 4.4%, *p* = 0.001) and Tjeldbergodden (R^2^ = 6.0%, *p* = 0.003) but not Solhomfjell (R^2^ = 2.8%, *p* = 0.080) (Table S4). Within Lund and Solhomfjell, ASV-level beta diversity was also significantly associated with DBH (Table S4). Within Tjeldbergodden, *Devosia*, *Gemmatimonas* and Candidatus *Udaeobacter* were associated with northern aspects compared to southern (Fig. 2). While unclassified at the genus-level, the abundance of the family Obscuribacteraceae (phylum Cyanobacteria) was also associated with northern aspects in Tjeldbergodden, as was unclassified members of the Cyanobacteria phylum (Fig. 2). In Lund, *Edaphobaculum* was associated with northern aspects compared to southern, while the opposite was true for *Acidisphaera* (Fig. 2). No genera were associated with aspect in Solhomfjell. No genera were associated with sampling height or DBH in any site.

**Table 1:** Results from PERMANOVAs on bacteria and green algae beta diversity at the genus- and ASV-levels

Variable	Degrees of freedom	Sum of squares	*R* ^2^	F statistic	*P* value
Bacteria: genus-level					
Plot	12	10,825	0.328	3.41	0.162
Aspect: south-north (cosine)	1	527	0.016	1.99	0.002
Aspect: west-east (sine)	1	246	0.007	0.93	0.097
Height above ground	1	240	0.007	0.90	0.697
Interaction: aspect (cosine) * site	2	752	0.023	1.42	0.250
Interaction: aspect (sine) * site	2	514	0.016	0.97	0.896
Residual	75	19,853	0.602	NA	NA
Total	94	32,957	1	NA	NA
Bacteria: ASV-level					
Plot	12	83,372	0.350	3.77	0.001
Aspect: south-north (cosine)	1	2915	0.012	1.58	0.001
Aspect: west-east (sine)	1	1822	0.008	0.99	0.173
Height above ground	1	1872	0.008	1.02	0.155
Interaction: aspect (cosine) * site	2	5707	0.024	1.55	0.001
Interaction: aspect (sine) * site	2	4381	0.018	1.19	0.398
Residual	75	138,069	0.580	NA	NA
Total	94	238,137	1	NA	NA
Green algae: genus-level					
Plot	12	1625	0.383	4.40	0.003
Aspect: south-north (cosine)	1	65	0.015	2.11	0.022
Aspect: west-east (sine)	1	30	0.007	0.97	0.319
Height above ground	1	43	0.010	1.40	0.144
Interaction: aspect (cosine) * site	2	85	0.020	1.38	0.143
Interaction: aspect (sine) * site	2	88	0.021	1.42	0.102
Residual	75	2311	0.544	NA	NA
Total	94	4248	1	NA	NA
Green algae: ASV-level					
Plot	12	7002	0.349	3.77	0.001
Aspect: south-north (cosine)	1	284	0.014	1.83	0.002
Aspect: west-east (sine)	1	181	0.009	1.17	0.102
Height above ground	1	149	0.007	0.97	0.826
Interaction: aspect (cosine) * site	2	481	0.024	1.55	0.002
Interaction: aspect (sine) * site	2	345	0.017	1.11	0.050
Residual	75	11,600	0.579	NA	NA
Total	94	20,041	1	NA	NA

### Green Algae Community

**Fig. 3:**
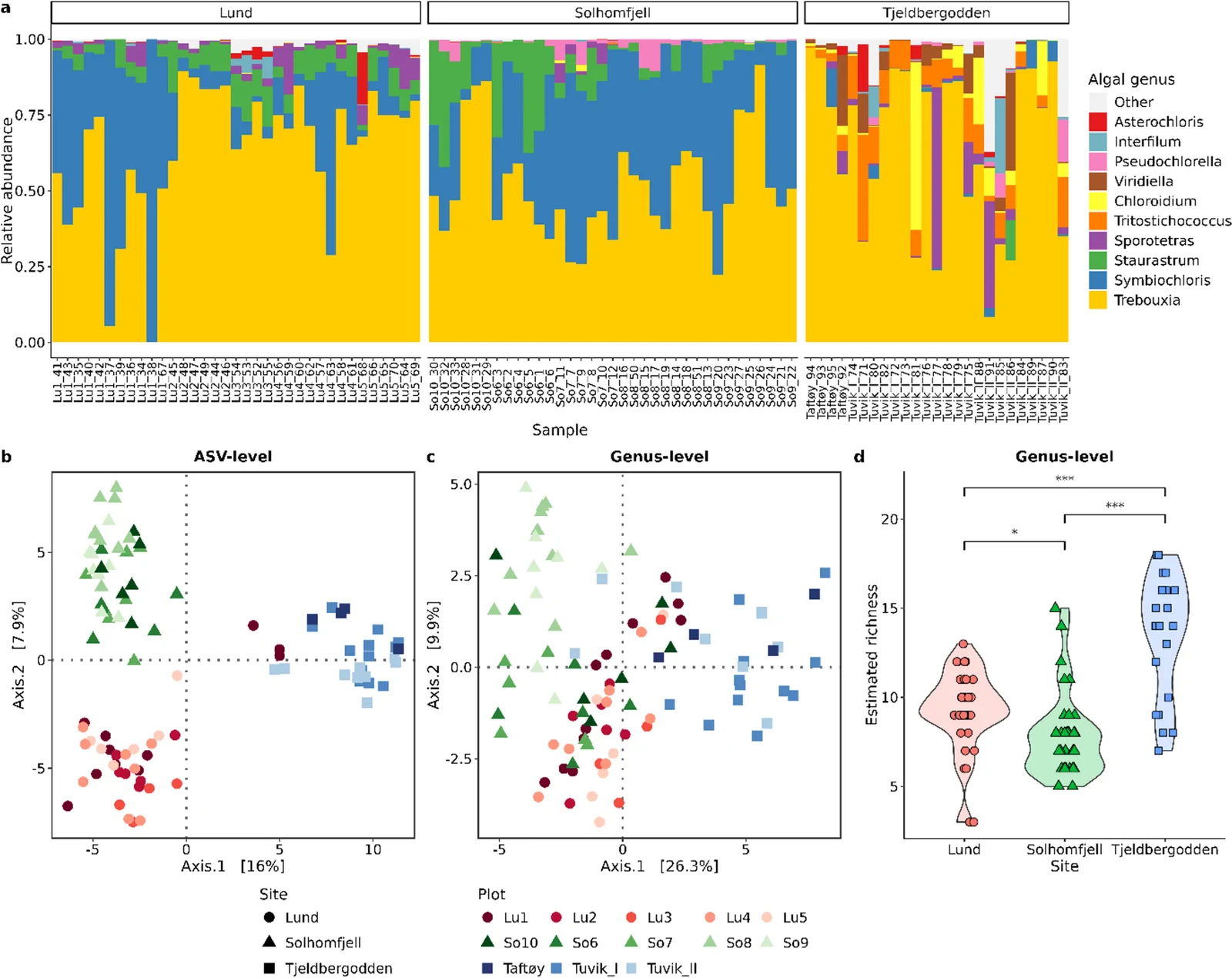
Green algae community composition differs by site. **a**) Relative abundance of the most abundant genera in each sample. Samples are grouped by site and ordered by plot, as indicated by the sample labels. The ten most abundant genera are shown, with all remaining genera grouped into ‘other’. **b–c)** PCoA of the community based on ASV abundances (**b**) and genera abundances (**c**), based on Aitchison distances from robust CLR transformed abundance values. Shapes indicate site while colours indicate plot. **d**) Violin plots of genus-level richness among sites. Statistically significant differences among sites are indicated by brackets (*p* < 0.001: ***; *p* < 0.01: **, *p* < 0.05: *)

In the ITS dataset, a total of 1,069 ASVs were identified and assigned to 31 green algae genera (Data S2). The algal community was dominated by the phylum Chlorophyta, specifically the order Trebouxiales. At the genus-level, *Trebouxia* was the most commonly detected genus in all sites, followed by *Symbiochloris* in Lund and Solhomfjell and *Tritostichococcus* in Tjeldbergodden (Fig. 3a). Genus-level richness significantly differed among sites, with Solhomfjell having slightly lower richness and Tjeldbergodden higher richness than Lund (Fig. 3d, Table S6). However, one plot in Solhomfjell (plot So7) had significantly higher richness than several others in that site (Fig. S7, Table S6). Beta diversity also significantly differed among sites and plots at the genus- and ASV-levels (Fig. 3b-c; Tables 1 and S4). Dispersions were significantly higher in Tjeldbergodden compared to Lund (*p* < 0.005), while dispersions among plots within each location were similar. Twelve genera significantly differed in relative abundance among sites, the majority of which belonged to the class Trebouxiophyceae (Table S7, Fig. S8).

**Fig. 4:**
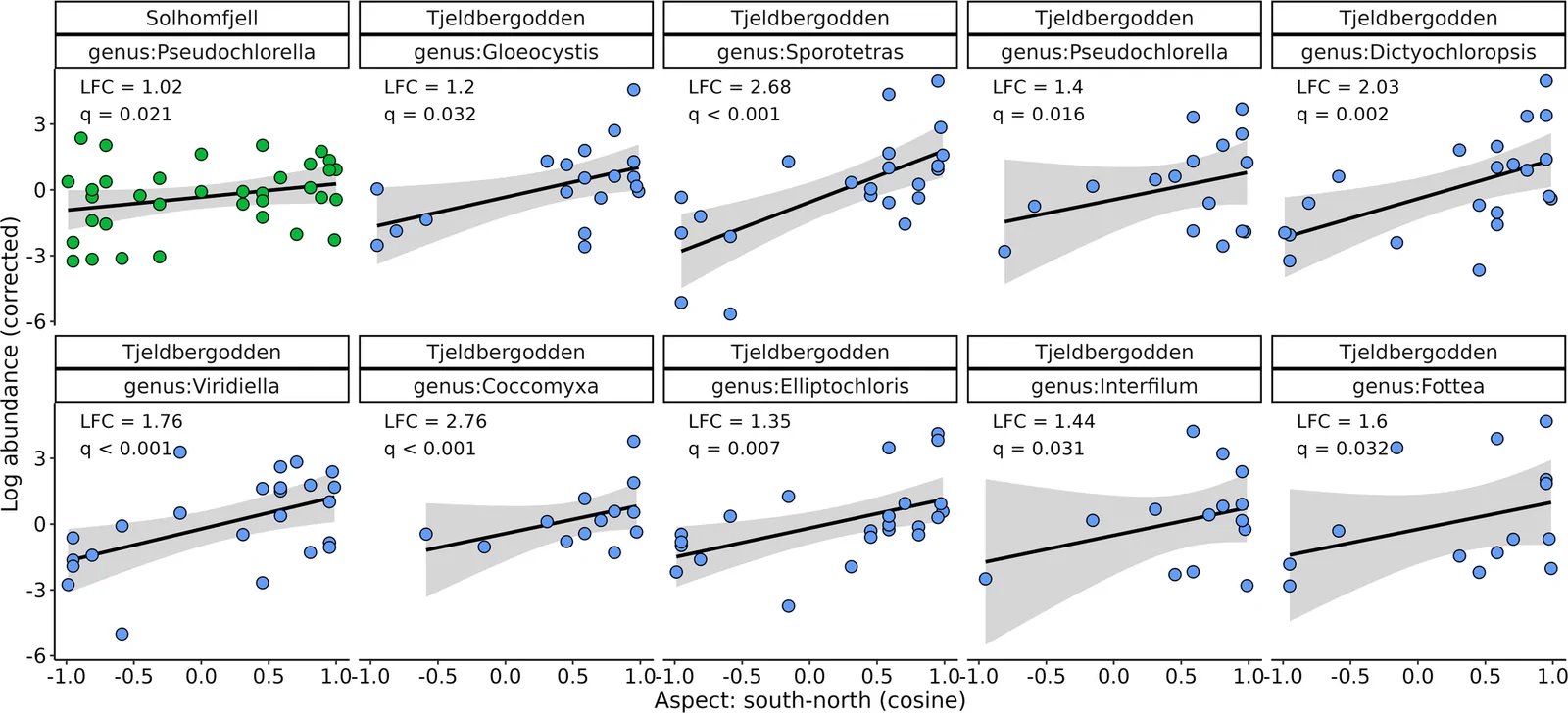
Algal genera associated with south–north aspects within Solhomfjell (green) and Tjeldbergodden (blue) sites (no taxa were associated with aspect in Lund). Algae abundance has been bias-corrected and log-transformed by ANCOM. Trend lines (black) with confidence intervals (grey shading) were generated using the lm method of the function geom_smooth within the R package ggplot2. The log-fold-change (LFC) and adjusted p value (q) for each ANCOM test is shown in the upper left corner of each plot

Within Tjeldbergodden and Lund, genus-level richness was positively associated with south–north aspects (Table S6). Sampling height and DBH were not associated with alpha diversity in any site. South–north aspect was associated with ASV-level beta diversity, an effect that varied among sites, explaining higher levels of variation in the data at Tjeldbergodden (R^2^ = 7.8%, *p* = 0.017) than Solhomfjell (R^2^ = 3.5%, *p* = 0.035) or Lund (R^2^ = 3.2%, *p* = 0.001) (Table S4). Within Tjeldbergodden, nine genera were associated with aspect, all with significantly higher relative abundances in northern compared to southern aspects (Fig. 4). The majority belonged to the Trebouxiophyceae class, including *Coccomyxa*, *Dictyochloropsis* and *Viridiella*. The genus *Pseudochlorella* (Trebouxiophyceae class) was positively associated with south–north aspect in both Tjeldbergodden and Solhomfjell, although the effect was stronger in the former (Fig. 4). None of the genera associated with aspect were differentially abundant among plots within a site. No genera were associated with aspect in Lund. No genera were associated with sampling height or DBH in any site.

### Field Observations vs. DNA-Based Relative Abundance of Cyanobacteria and Photosynthetic Eukaryotes

**Fig. 5:**
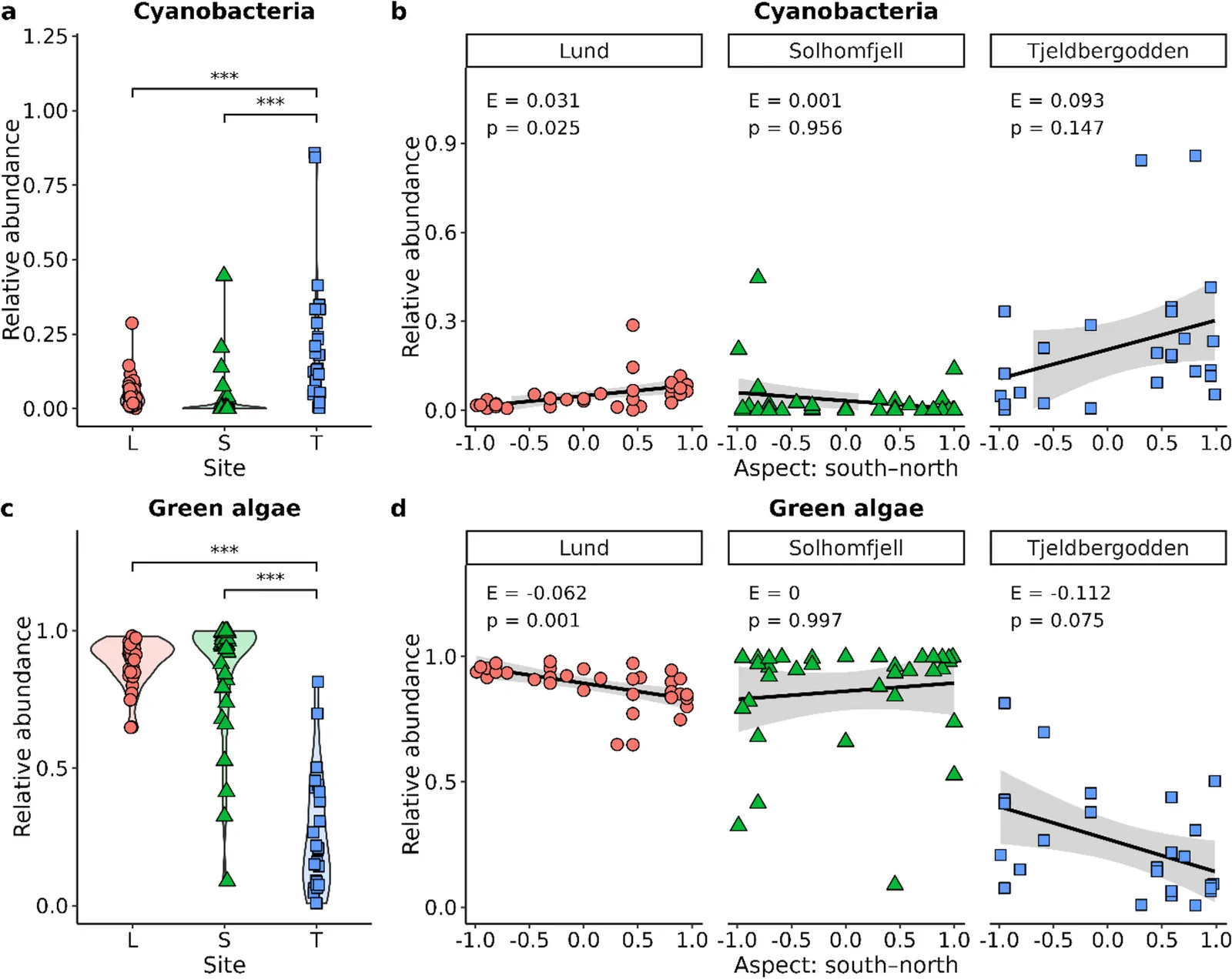
Relative abundance of cyanobacteria (**a–b**) and green algae (**c–d**). **a**, **c**) Differences among sites. Statistically significant differences from pairwise comparisons between sites are indicated by brackets (*p* < 0.001: ***). L: Lund, S: Solhomfjell, T: Tjeldbergodden. **b**, **d**) Differences in along south–north aspect (transformed using cosine) within each site. Trend lines with confidence intervals (grey shading) were generated using the lm method of the function geom_smooth within the R package ggplot2. The slope (E) and p value (p) for each linear model is shown in the upper left corner of each plot. Relative abundance was calculated from the number of 16S reads assigned to cyanobacteria or green algae, divided by the total number of 16S reads assigned to cyanobacteria and photosynthetic eukaryotes

We reanalysed the field data from 2011 to 2020, confirming a significant increase in aerophytic algae cover at Lund compared to Tjeldbergodden (*p* < 0.05) and a significant increase in bryophytes at Tjeldbergodden compared to Lund or Solhomfjell (*p* < 0.05) (Fig. S9). Crustose lichen cover was also lower at Lund compared to the other two sites (Fig. S10, *p* < 0.05). To compare to the metabarcoding results from samples collected in 2022, we used Genbank taxonomic assignments of 16S chloroplast reads to estimate the relative abundance (proportion) of cyanobacteria and photosynthetic eukaryotes (Data S3). The abundance of bryophytes was higher in Tjeldbergodden (Fig. S11, Table S8) and significantly correlated with field observations of bryophytes from 2011 (Fig. S9, ρ = 0.609, *p* = 0.027). The relative abundance of cyanobacteria was significantly higher in Tjeldbergodden compared to Lund or Solhomfjell (Fig. 5a), while the relative abundance of green algae was significantly lower (Fig. 5c, Table S8). There was good agreement between the field observations of aerophytic algae from Tjeldbergodden and Lund in 2011 and 2016, respectively, and the relative abundance of green algae from 2022; however, field observations from Solhomfjell reported lower levels of aerophytic algae in 2020 than the metabarcoding results from 2022 (Fig. S9). The relative abundance of cyanobacteria tended to increase in northern aspects compared to southern aspects for both Tjeldbergodden and Lund (Fig. 5b), while the relative abundance of green algae tended to decrease (Fig. 5d, Table S8). Within Lund, one plot (Lu4) had significantly lower abundance of green algae compared to the other plots and within Solhomfjell, one plot (So8) had significantly higher abundance of cyanobacteria than the other plots (Fig. S12, Table S8). Within this plot in Solhomfjell, DBH was associated with higher abundance of cyanobacteria (Fig. S13).

### Network Analysis

**Fig. 6:**
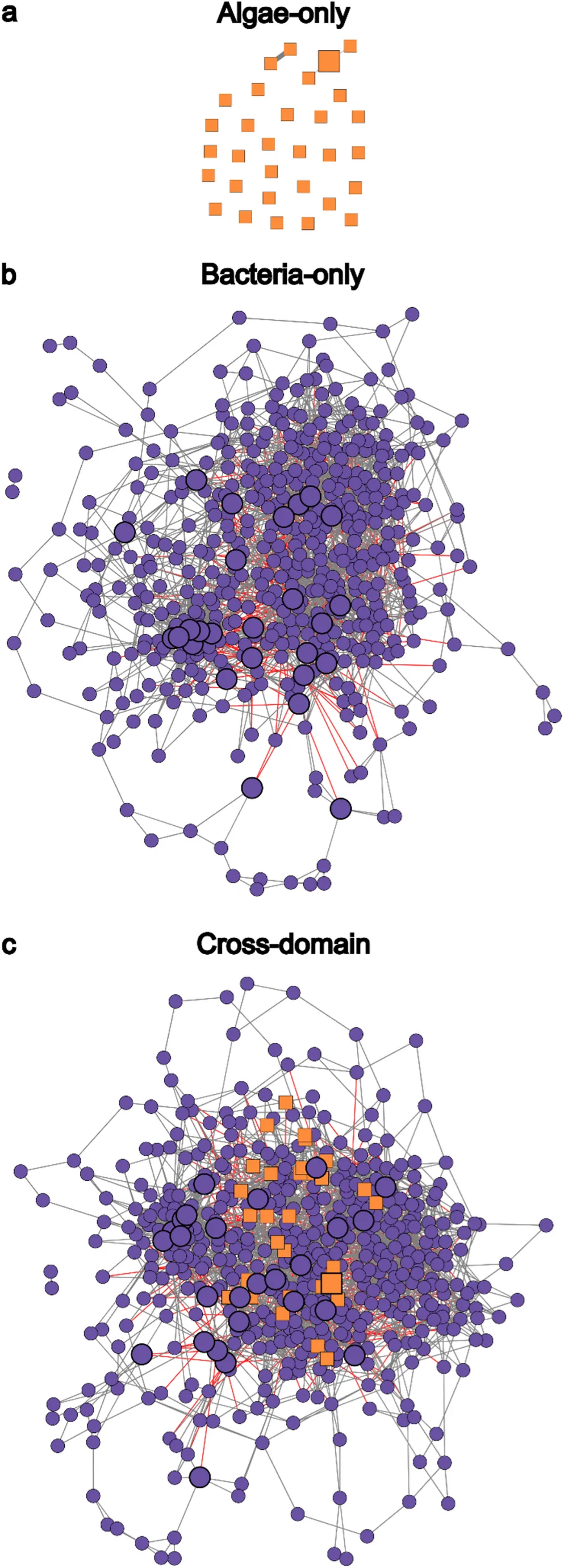
Genus-level co-occurrence networks of green algae (**a**), bacteria (**b**) and the cross-domain network of algae and bacteria (**c**). Each purple circle node represents a bacterial genus and each orange square node represents an algal genus. Hub nodes are highlighted with thick black borders and are slightly larger than non-hub nodes. Edges in grey indicate a positive correlation between two nodes, with darker and thicker grey edges indicating a larger correlation. Edges in red indicate a negative correlation between two nodes

In the algae-only co-occurrence network (Fig. 6a), five of the 31 genera formed two small clusters, while the remaining 26 (83%) were not connected to any other taxa (‘singletons’). *Pseudendoclonium* and *Vulcanochloris* were strongly positively associated in one cluster, while in the other, *Desmococcus* formed a hub node with weaker positive associations to *Apatococcus* and *Myrmecia*. The algal network was thus very sparely connected, with a clustering coefficient of 0, an average path length of 0.194 and a modularity of 0.444 (Table 2). Observed network properties were not significantly different from random networks of the same density (Table 2).

In the bacteria-only co-occurrence network, the 503 genera formed 9 clusters, with 26 highly connected hub taxa and no singletons (Fig. 6b). The network was much more densely connected than the algae-only network, with a clustering coefficient of 0.202, an average path length of 2.390 and a modularity of 0.491 (Table 2). All evaluated network properties were significantly higher (*p* < 0.001) in the observed network compared to random networks of the same density (Table 2). Cluster 6 (105 genera) included the most abundant bacterial genera (114-901-12, *Granulicella* and *Acidiphilium*) and several bacteria associated with differential abundance among sites, including hub taxa *Jatrophihabitans* and *Sphingomonas*. Cluster 4 (59 genera) and cluster 7 (146 genera) also included several bacteria associated with differential abundance among sites.

In the cross-domain network, the 503 bacterial and 31 algal genera formed 11 clusters, with no unconnected taxa (Fig. 6c). The cross-domain network was similar to the bacterial-only network, with a clustering coefficient of 0.200, an average path length of 2.324 and a modularity of 0.490 (Table 2). Domain-level assortativity was moderately high at 0.456, indicating that nodes from the same domain tended to cluster together. All evaluated network properties were significantly higher (*p* < 0.001) than expected in a random network (Table 2). Twenty-seven hub taxa were identified in eight clusters, including 23 bacteria that were also hubs in the bacterial-only network, three new bacterial hubs and one algal hub (*Chloroidium*). Of the 11 clusters, three clusters had at least 2 algal members and > 20 bacterial members (cluster 1, 6 and 8). The remaining clusters either had only 1 algal member and many bacterial members (cluster 2, 9 and 10) or were composed of only bacteria. We hereafter focus on the cross-domain clusters. Cluster 1 (82 bacteria, 12 algae) included the most abundant bacterial and algal genera (e.g. 114-901-12 and *Trebouxia*) and many taxa with significantly different abundance among sites, including the algal hub *Chloroidium* and bacterial hubs *Jatrophihabitans* and *Sphingomonas*. Cluster 6 (22 bacteria, 12 algae) included all connections observed in the algae-only network (e.g. *Apatococcus* and *Desmococcus*). Cluster 8 (135 bacteria, 4 algae) included several taxa associated with differential abundance among sites and/or associated with aspect, including several with increased abundance in northern aspects of Tjeldbergodden (e.g. *Devosia* and *Coccomyxa*).

**Table 2:** Comparison of properties between the single-domain and cross-domain networks

Network property	Algae-only	Bacteria-only	Cross-domain
Diameter ^2^	2	11*	9*
No. of clusters	2	9	11
No. of taxa in clusters	5	503	534
No. of singleton taxa	26	0	0
Clustering coefficient ^1^	0	0.202	0.200
Transitivity ^2^	0	0.273*	0.259*
Modularity ^1^	0.444	0.491*	0.490*
% of positive edges ^1^	100	82.062	80.107
Network density ^1^	0.006	0.018	0.018
Natural connectivity ^2^	0.004	0.037*	0.035*
Average dissimilarity ^1^	0.998	0.994	0.994
Average path length ^1^	0.880	2.390*	2.324*
Average node degrees ^1^	0.194	9.177	9.772
Assortativity (domain-level) ^2^	NA	NA	0.456*

^1^ Calculated via NetCoMi

^2^ Calculated via igraph

* Significantly higher than null model (*p* < 0.001)

## Discussion

The microbial community of Norwegian birch bark was dominated by similar taxa across the three sites, including genera of the bacterial orders Acetobacteriales, Acidobacteriales and Rhizobiales (now Hyphomicrobiales) and the aerophytic algal order Trebouxiales. Such taxa have also been found to dominate the bark communities of several other European tree species [3, 8, 10, 11, 14–16]. This consistency suggests a conserved component of the microbial community that is well-adapted to the general bark environment and may perform important ecological functions. While many functions of corticolous microbes are unknown, a recent metagenomics study of Australian trees identified several bark-associated bacteria that metabolise methane and hydrogen, contributing to global biogeochemical cycles [4]. Rhizobiales/Hyphomicrobiales are nitrogen-fixing bacteria that form symbiotic relationships with plant roots [62], and therefore may perform similar functions on tree bark [16]. They have also been associated with the microbiota of epiphytic lichens [8]. Similarly, algae of the order Trebouxiales are frequently found on tree bark [3, 14–16] and also form associations with epiphytic lichens [63]. While we aimed to sample bark free of lichens and other epiphytes, a previous study has documented overlap between lichen- and moss-associated bacterial communities and bacteria on bare bark, suggesting that bacteria may be transferred between the three habitats [8]. Thus, some of the bacteria and algae we observed may be derived from or play a role in associations with lichens and other epiphytes [3].

Despite the observed similarities among sites, there were nevertheless many genera with abundances that significantly differed among sites. However, due to the lack of knowledge on corticolous microbial functions and the limitations of the 16S gene for bacterial functional inference [64], we were unable to identify any putative functional roles of these taxa. Genus-level alpha diversity was significantly higher at Tjeldbergodden than Lund or Solhomfjell. Tjeldbergodden had the greatest diversity in epiphytes, including foliose and crustose lichens, mosses and liverworts. The increased epiphytic diversity at Tjeldbergodden may have provided additional microbial niches and nutrients, resulting in more diverse communities even on the sampled bare bark [8]. Beta diversity, particularly at the ASV-level, also demonstrated clear differentiation among sites, suggesting that site specificity is driven by the presence of strain-level variation. These results suggest that corticolous microbial communities structure at different ecological scales than the dominant vegetation communities, which were similar at each site. The site-specific microbes may be adapted to local environmental conditions, such as differences in humidity, temperature or light availability, or seasonal fluctuations [9–11, 15, 17]. While our sites generally had similar climates, Lund and Solhomfjell were sampled in summer while Tjelbergodden was sampled in late autumn, generally characterised by cooler temperatures, less daylight and increased precipitation. Such environmental differences may have contributed to the increased inter-sample variation observed at Tjeldbergodden. Environmental variables, such as temperature, were not collected at the time of sampling, thus we were unable to investigate these effects further.

One objective of this study was to compare the corticolous microbial community at sites with differing histories of nitrogen deposition. Atmospheric deposition of reactive nitrogen compounds has decreased at all three sites over the past 30 years [28–30]. While nitrogen availability was not measured at the time of sampling, historical nitrogen deposition has been demonstrated to have had cumulative, persistent impacts in terrestrial ecosystems [65–67]. In this study, we focused on these indirect, legacy effects of nitrogen deposition, by focusing on microorganisms that are likely important to nitrogen cycling in bark habitats. Estimated nitrogen deposition was three-fold higher in Lund compared to Tjeldbergodden in the five years before sampling (Table S1). Despite the many years between in sampling, both observations from the earlier field survey and the more recent metabarcoding found that non-diazotrophic green algae were at higher abundance at Lund, while metabarcoding revealed a decreased abundance of cyanobacteria. The reverse pattern was observed at Tjeldbergodden. These findings are consistent with previous studies, where nitrogen deposition has been found to promote the growth of non-diazotrophic green algae [24, 25], reflecting the situation at Lund, while nitrogen-fixing cyanobacteria have a competitive advantage in nitrogen-limited environments [53–55], like that at Tjeldbergodden. While these results are consistent with a persistent, legacy effect of nitrogen deposition, it must be noted that there are limitations to using the 16S gene as a marker for abundance of photosynthetic eukaryotes. Differences in organism copy number, primer biases towards prokaryotes, and differences in DNA extraction efficiency of eukaryotic versus prokaryotic cells can impact abundance estimates, and as such, our results should be interpreted with caution. More research is needed to understand the effects of pollution-derived compounds on corticolous microbial communities. Future studies could be benefited by incorporating environmental variables (such as temperature), pollutant concentrations and quantified microbial biomass into models of microbiota variation.

A second objective of this study was to evaluate the effect of tree-specific microscale factors on the microbial communities of bark, focusing specifically on aspect on the trunk. In the northern hemisphere, north-facing aspects are expected to favour microbes with preferences for damper or darker conditions, due to reduced solar radiation compared to south-facing aspects [11, 15, 17, 68]. Our results add support to previous findings that the effect of aspect is site-dependent. We observed associations between northern vs. southern aspect and alpha and beta diversity in the algal and bacterial communities at Lund and Tjeldbergodden, but not at Solhomfjell. We also found higher abundances of cyanobacteria at northern compared to southern aspects in Lund and Tjeldbergodden, while in Tjeldbergodden, several other bacterial and algal taxa had significantly higher abundances at northern aspects. Previous studies have found that the effect of aspect is stronger on solitary trees or more open landscapes, compared to continuous forest, where neighbouring trees may homogenise sunlight exposure [11, 15, 68]. For example, cyanobacteria have been found at higher abundances on northern aspects of clear-cut aspen stands compared to southern aspects, while the effect of aspect was weaker in forest stands [68]. Trees at two of the three Tjeldbergodden plots were sampled from small stands in coastal heathland (Tuvik I and II), whereas sampling generally occurred in continuous forest at the other sites, thus increased landscape openness may contribute to the stronger effect of aspect at Tjeldbergodden. However, as only four samples were taken from the mature forest site at Tjeldbergodden (Taftøy), we did not have the statistical power to test this hypothesis. Overall, our results suggest that microhabitat factors like aspect could act as environmental filters for bark microbiota, mediated by macrohabitat factors like landscape openness. Such shifts in taxonomic composition may be associated with changes in ecological functions in the birch bark microbial habitat. For example, increases in nitrogen-fixation by cyanobacteria at northern aspects of small birch stands may provide bioavailable nitrogen to other members of the bark ecosystem, like non-diazotrophic green algae [69]. Future metagenomic and metatranscriptomic studies could provide better insights into the functional consequences of these taxonomic changes.

By characterising both the bacterial and the algal communities, we were able to investigate cross-domain associations via co-occurrence networks. We found that the algal community, in the absence of bacteria, formed a sparse, disconnected network with low robustness. In the cross-domain network, algal taxa were generally interspersed among bacteria-dominated clusters, with a network structure that was similar to the bacteria-only network and significantly different from that expected at random. These results suggest that in the birch bark microbiota, algae form specialised niches, connected by a backbone of more generalist bacteria. Similar findings have been observed between bacteria and algae (and fungi) in the beech bark microbiota [16], providing evidence for a conserved higher-level corticolous microbiota structure across multiple European tree species. The large percentage of positive associations observed in the cross-domain network (ca. 80%) may represent mutualistic interactions between bacteria and algae, such as through nutrient sharing [8, 69]. For example, we identified the bacterial genus *Sphingomonas* as a highly connected hub taxon in a cross-domain cluster that also contained algal genera *Trebouxia* and *Symbiochloris* (order Trebouxiales). *Sphingomonas* has been previously identified as a facultative photosynthetic generalist on tree bark [8] that associates with Trebouxiales algae in cross-domain networks [16]. *Trebouxia* and *Symbiochloris* are both primary producers which likely support bacterial communities, which together with fungi form important components of tree-associated food webs for microarthropods and microscopic metazoans [3]. However, as our results are based on co-occurrence data, we cannot identify causal ecological interactions from our dataset. Indeed, some of the cross-domain network structure likely reflects environmental conditions affecting both the bacterial and algal communities, since several clusters contained algal and bacterial genera with differential abundances associated with site or aspect. Similar to our results, corticolous bacterial and algal communities have been found to co-vary with environmental factors in previous studies [10]. Further work is needed to investigate interactions among the various members of tree bark communities, including their functional roles and their responses to changing environmental conditions.

In this study, we provide the first characterisation of the bark-associated microbial community of birch, one of the most common and ecologically important trees in Norway [27, 28]. Consistent with previous work in other tree species, we find that the birch corticolous microbiota consists of a robust network of bacteria interspersed by specialised, phototrophic algae. Our results suggest that microhabitat and spatial context, such as reduced solar radiation, landscape openness and other site-dependent variation, may shape the taxonomic composition of the birch bark microbiota. We hypothesise that such changes may have functional consequences for the bark ecosystem, for example, by changing the abundance of primary producers and nitrogen-fixers, with possible effects on the broader tree-associated food web. However, more research is needed to elucidate the ecological roles of the bark-associated microbiota, including their interactions with each other, other epiphytes, the host tree and the surrounding forest system. 

## Supplementary Information

Below is the link to the electronic supplementary material.

Supplementary Material 1

Supplementary Material 2

## Data Availability

Sequencing data are publicly available on the European Nucleotide Archive (ENA) under project PRJEB109031. The amplicon sequence variants generated as part of this project, including taxonomy assignment to the genus-level and DNA sequence, are available in Data S1–3.
