# Supplementary material for: Bacterial and Green Algal Communities of Norwegian Birch Tree Bark
Source: Microb Ecol. 2026 Jul 21;89:188. doi: 10.1007/s00248-026-02841-z (PMC13672728; doi:10.1007/s00248-026-02841-z)
Supplement: Supplementary file 1 [file 248_2026_2841_MOESM2_ESM.pdf]

Bacterial and green algal communities of Norwegian birch tree bark

Jaelle C. Brealey[1], Marie L. Davey[1], Bård Pedersen[1]

[1] Land and biodiversity, Norwegian Institute for Nature Research, Trondheim, Norway

Corresponding author: Jaelle Brealey, jaelle.brealey@nina.no

Supplementary Material

Supplementary methods 3

Selection of sampling plots 3

Selection of bark samples 3

DNA extraction 3

PCR of target genes 3

Processing of amplicon sequences 4

Reference list 4

Supplementary data 5

Data S1. Bacteria 16S ASVs, taxonomy and number of occurrences in each site 5

Data S2. Green algae ITS ASVs, taxonomy and number of occurrences in each site 5

Data S3. 16S ASVs of cyanobacteria and photosynthetic eukaryotes, taxonomy and number of occurrences in each site 5

Supplementary figures 6

Fig. S1. Sampling site location and site characteristics 6

Fig. S2. Composition of the epiphyte community on birch trunks 7

Fig. S3. Violin plots showing variation in measured covariates by site and plot 8

Fig. S4. Relative abundance of the most bacterial abundant genera in each sample 9

Fig. S5. The three most common bacterial genera associated with site 9

Fig. S6. The 14 bacterial genera most strongly associated with site 10

Fig. S7. Violin plots of algae genus-level richness among plots within each site 11

Fig. S8. All 12 green algae genera significantly associated with site 12

Fig. S9. Observations from the field survey of epiphytes compared to the abundance of photosynthetic eukaryotes detected via 16S metabarcoding 13

Fig. S10. Observations from the field survey of epiphytic lichens 13

Fig. S11. Proportion of 16S sequences assigned to bryophytes 14

Fig. S12. Proportion of 16S sequences assigned to cyanobacteria (**A**) and green algae (**B**) by plot within each site 15

Fig. S13. Proportion of 16S sequences assigned to cyanobacteria by tree size (diameter at breast height, DBH) within each plot in Solhomfjell 16

Supplementary tables 17
Table S1. Characteristics of sampled sites, including climate and vegetation zones, estimated annual S- and N deposition and epiphyte communities 17
Table S2. Location, elevation, field layer vegetation and birch stand characteristics of sampled plots 18
Table S3. Results from linear models of bacterial alpha diversity (genus-level richness)...... 19
Table S4. Results from PERMANOVAs on bacteria and green algae beta diversity at the genus- and ASV-levels within each site 20
Table S5. Bacteria genus-level differential abundance results from ANCOM pairwise comparisons of site 24
Table S6. Results from linear models of green algal alpha diversity (genus-level richness)... 30
Table S7. Algae genus-level differential abundance results from ANCOM pairwise comparisons of site 31
Table S8. Results from linear models of relative abundance of bryophytes, cyanobacteria and green algae 32

Supplementary methods

Selection of sampling plots

All plots in the present study were part of a previously completed epiphyte monitoring program (1–3). In this program, circular plots with a radius of 10 meters were selected based on the following inclusion criteria: each plot had to contain at least 10 birch trees with circumferences at breast height (1.3 m) larger than 30 cm, with intact main branches and tops. Additionally, plots were preferably dominated by bilberry (*Vaccinium myrtillus*) in the field layer (Table S2).

Selection of bark samples

Eight birch trees with circumferences larger than 30 cm were randomly selected in each plot as candidate trees for sampling. Sampling was limited to surfaces where the monitoring program had documented the presence of epiphytes, and, for practical reasons, to heights on the trunk section 130–185 cm above ground level, corresponding to the lower part of the bark surface covered by the monitoring program. We divided the radial gradient into eight sectors, each covering an equal interval of aspects, i.e. 45°. Before the field campaign, five (Lund, Solhomfjell) or four (Tjeldbergodden) candidate sampling points were randomly drawn from each sector and locality, regardless of plot and tree, from the potential points satisfying the above criteria. A larger, stratified, random sample of reserve points were drawn for each site. In the field, pre-selected sampling points were replaced with reserve points from the same site and sector if: 1) there were no visible epiphytes, 2) sampling failed due to mishaps, or 3) sampling could not be done for practical reasons, e.g. branches in the immediate vicinity. In Tjeldbergodden, we ran out of reserve points, hence only 25 samples were used in the analysis (compared to 35 for Lund and Solhomfjell).

DNA extraction

DNA extractions and library construction were carried out at the Center for Biodiversity Genetics (NINAGEN) at NINA. To ensure extraction of only the epiphytic algal and bacterial communities, bark samples were thawed prior to DNA extraction, and the surface was sampled by rolling a nylon flocked swab with a 30 mm breakpoint moistened with molecular grade water over the entire surface area of the bark sample, excluding any area occupied by epiphytic lichens or mosses. The swab was then deposited into 1 mL of ATL buffer (Qiagen) contained in a 2mL Lysing Matrix E tube (MPBio; contains 1.4 mm ceramic spheres, 0.1 mm silica spheres, and eight 4 mm glass beads). Each subsample was initially homogenised using a FastPrep-24™ instrument (MP Biomedicals) at 6.0 m/s for 40 s, before adding 100 µl of Proteinase K and incubating at 56 °C overnight. The samples were then homogenised twice more using the FastPrep-24™ instrument as described above, and DNA was extracted from 300 µl of each homogenate using the KingFisher Tissue kit (ThermoFisher Scientific) as per the manufacturer's instructions. The DNA was eluted in 150 µl Buffer AE (Qiagen; 10 mM Tris-CL, 0.5 mM EDTA). A negative control (buffer only) was included for homogenisation and extraction.

PCR of target genes

The following primers were used to target the v4 region of the bacterial 16S rDNA gene (4,5):
forward 515F: 5'-GTGYCAGCMGCCGCGGTAA-3'
reverse 806R: 5'-GGACTACNVGGGTWTCTAAT-3'.

The following primers were used to target the green algae ITS2 gene (6):
forward ITS-Cha3: 5'-CAACTCTCRRCAACGGATA-3'
reverse ITS-u4: 5'-RGTTTCTTTTCCTCCGCTTA-3').

PCR reactions contained KAPA HiFi HotStart ReadyMix (Roche, Switzerland), 0.2 µM of the 16S primers or 0.4 µM of the ITS primers and 12.5 ng template DNA with a final volume of 25 µl. PCR

conditions for the 16S primers consisted of an initial denaturing step of 3 min at 94 °C, followed by 35 cycles of 45 s at 94 °C, 60 s at 50 °C and 90 s at 72 °C, followed by a final elongation step of 10 min at 72 °C. PCR conditions for the ITS primers consisted of an initial denaturing step of 5 min at 95 °C, followed by 30 cycles of 15 s at 95 °C, 15 s at 54 °C and 10 s at 72 °C, followed by a final elongation step of 10 min at 72 °C. PCR product quantity and quality were determined with the Nanodrop (ThermoFisher Scientific) and TapeStation 4200 systems (Agilent).

Processing of amplicon sequences
Primer sequences were removed from the forward and reverse reads using cutadapt v3.7, with a minimum length of 17, a mismatch rate (-e) of 0.15 and otherwise default parameters (7). Sequences were then processed in R v4.4.1 (8) using the pipeline DADA2 v1.32.0 (9). Quality filtering parameters included the truncation of sequences at the first base with a PHRED score < 10 and the removal of sequences with ambiguous bases, > 2 expected errors in the forward or reserve reads, a PHRED base score < 10 after trimming or a length < 150 bp after trimming. For the bacterial 16S dataset, longer forward and reverse sequences were also hard trimmed to 230 bp, after reviewing quality score profiles; this trimming was not required for the algal ITS dataset. Error rates were estimated by enforcing monotonicity for both the forward and reverse reads, to account for the binned error rates returned by the Illumina NovaSeq platform. Amplicon sequence variants (ASVs) were inferred for each sample and forward and reverse reads were merged with a minimum overlap of 30 bp. Chimeric sequences were evaluated on a per-sample basis and were removed if any ASV was flagged as chimeric in more than 90% of the samples (the "consensus" method).

Reference list

1. Framstad E. Terrestrial Ecosystems Monitoring in 2020: Ground vegetation, epiphytes, small mammals and birds. Summary of results [Internet]. 148. Norsk institutt for naturforskning (NINA); 2021 [cited 2025 Oct 9]. (NINA Report). Report No.: 1972. Available from: https://brage.nina.no/nina-xmlui/handle/11250/2734736

2. Framstad E. Terrestrial Ecosystems Monitoring in 2016: Ground vegetation, epiphytes, small mammals and birds. Summary of results [Internet]. 122 s. Norsk institutt for naturforskning (NINA); 2017 [cited 2025 Oct 9]. (NINA Report). Report No.: 1376. Available from: https://brage.nina.no/nina-xmlui/handle/11250/2452619

3. Pedersen B, Aarrestad PA. Program for environmental monitoring Tjeldbergodden. Monitoring of soil, vegetation and epiphytes 2011 [Internet]. 113. Norsk institutt for naturforskning; 2012 [cited 2025 Oct 9]. (NINA Report). Report No.: 861. Available from: https://brage.nina.no/nina-xmlui/handle/11250/2643028

4. Apprill A, McNally S, Parsons R, Weber L. Minor revision to V4 region SSU rRNA 806R gene primer greatly increases detection of SAR11 bacterioplankton. Aquatic Microbial Ecology. 2015 Jun 4;75:129–37.

5. Caporaso JG, Lauber CL, Walters WA, Berg-Lyons D, Lozupone CA, Turnbaugh PJ, et al. Global patterns of 16S rRNA diversity at a depth of millions of sequences per sample. Proceedings of the National Academy of Sciences. 2011;108:4516–22.

6. Dreyling L, Schmitt I, Dal Grande F. Tree Size Drives Diversity and Community Structure of Microbial Communities on the Bark of Beech (Fagus sylvatica). Frontiers in Forests and Global Change [Internet]. 2022 [cited 2024 Jan 10];5. Available from: https://www.frontiersin.org/articles/10.3389/ffgc.2022.858382

7. Martin M. Cutadapt removes adapter sequences from high-throughput sequencing reads. EMBnet.journal. 2011 May 2;17(1):10–2.

8. R Core Team. R: A Language and Environment for Statistical Computing [Internet]. Vienna, Austria: R Foundation for Statistical Computing; 2020. Available from: https://www.r-project.org/

9. Callahan BJ, McMurdie PJ, Rosen MJ, Han AW, Johnson AJA, Holmes SP. DADA2: High-resolution sample inference from Illumina amplicon data. Nat Methods. 2016;13(7):581–3.

Supplementary data

Data S1. Bacteria 16S ASVs, taxonomy and number of occurrences in each site. Please see attached Excel file.

Data S2. Green algae ITS ASVs, taxonomy and number of occurrences in each site. Please see attached Excel file.

Data S3. 16S ASVs of cyanobacteria and photosynthetic eukaryotes, taxonomy and number of occurrences in each site. Please see attached Excel file.

Supplementary figures

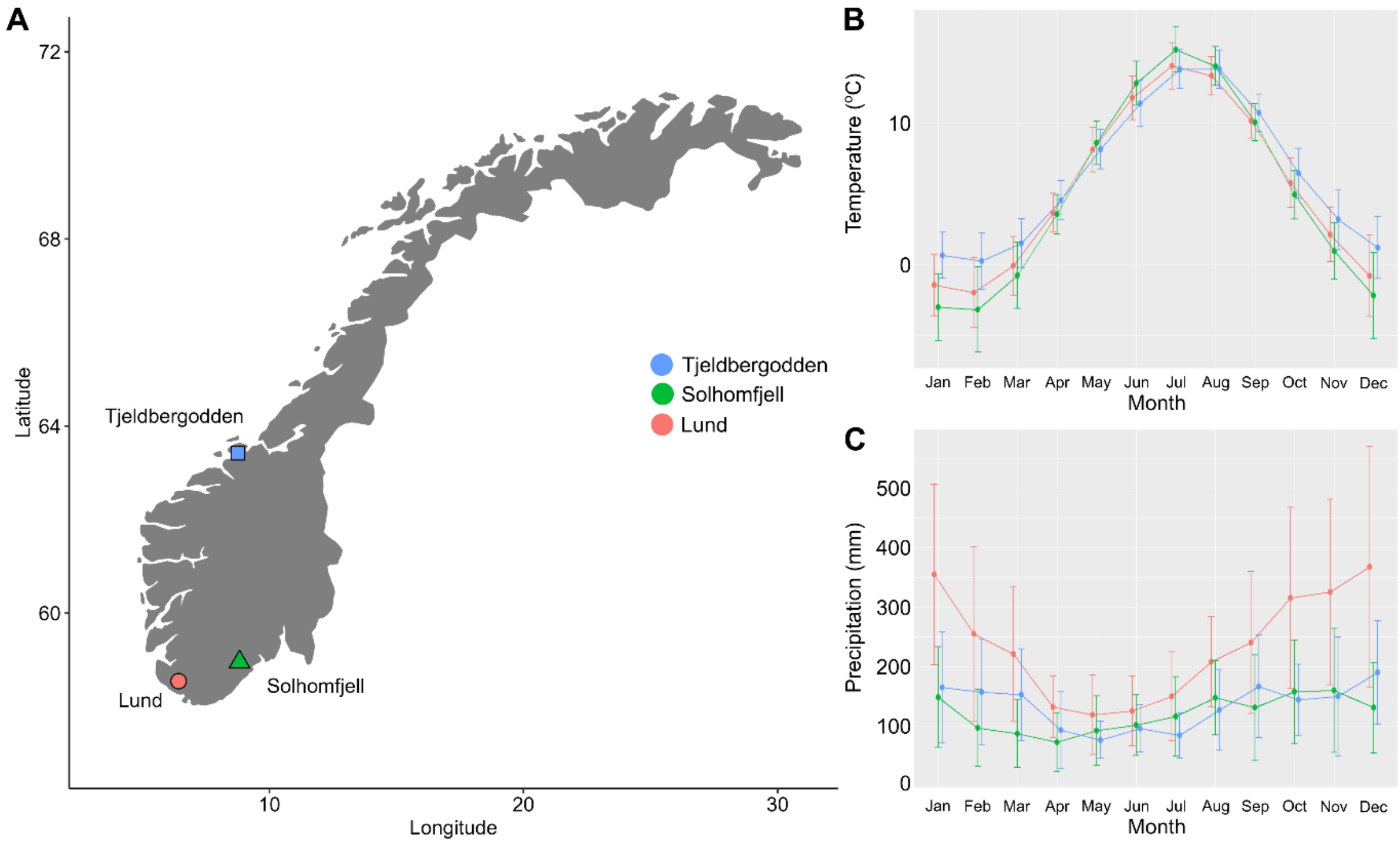

Fig. S1. Sampling site location and site characteristics. **A**) Map of Norway showing the location of Tjeldbergodden in central Norway and Solhomfjell and Lund in southern Norway. **B**). Monthly mean temperatures at each site. **C**). Monthly total precipitation at each site. In B-C, the mean ± SD for the period 1991–2020 are shown. Meteorological data was downloaded from the Norwegian Meteorological Institute (April 23, 2021) from the following sources: Lussana, C. 2020. seNorge observational gridded dataset. seNorge_2018, version 20.05. MET report No. 07/2020. https://www.met.no › MET-report-07-2020; Norwegian Meteorological Institute. 2021. Norwegian observational gridded climate datasets [Data set]. Thredds.met. https://thredds.met.no/thredds/catalog/senorge/seNorge_2018/Archive/catalog.html.

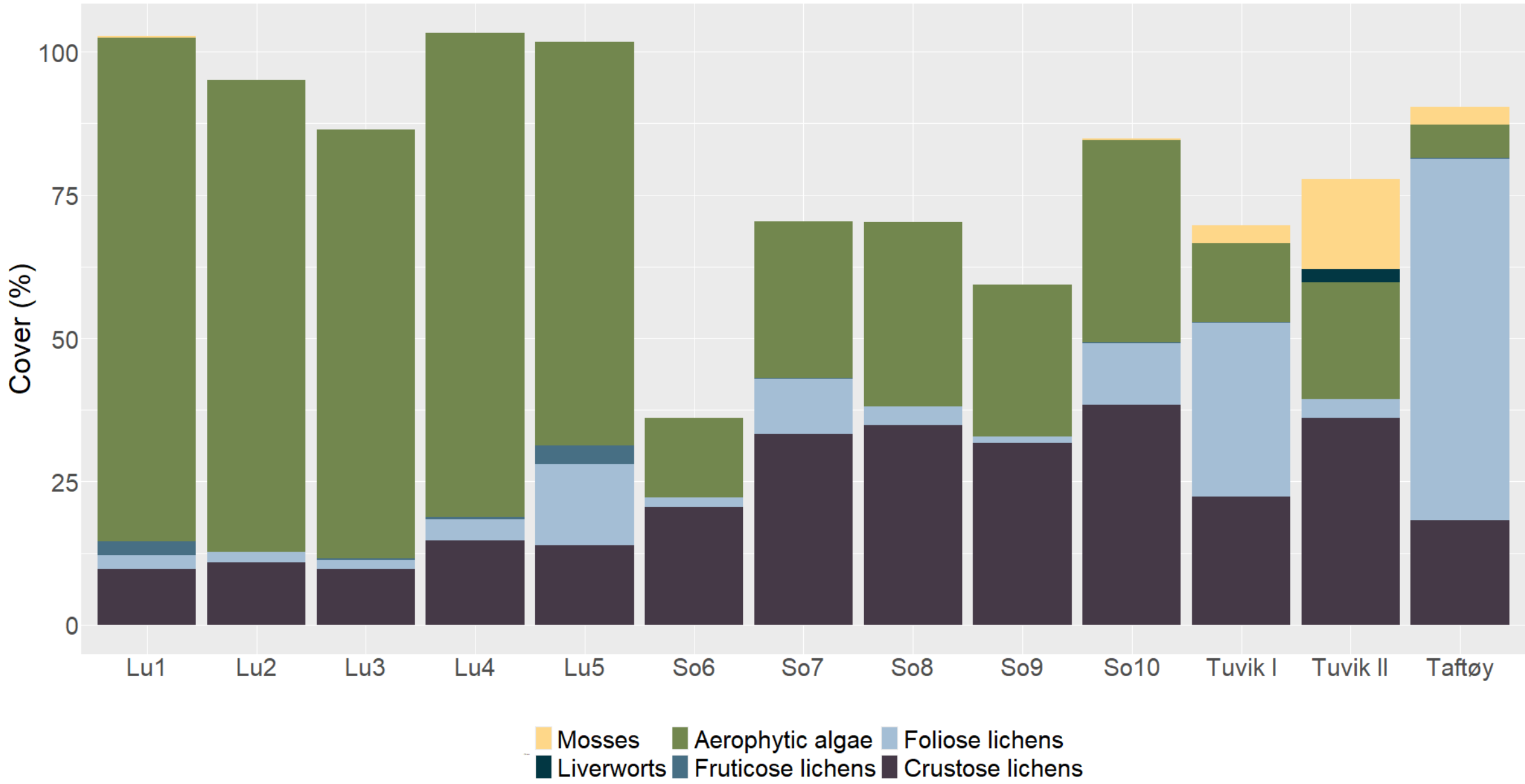

Fig. S2. Composition of the epiphyte community on birch trunks at each plot in Lund (Lu1-5), Solhomfjell (So6-10) and Tjeldbergodden (Tuvik I, Tuvik II and Taftøy).

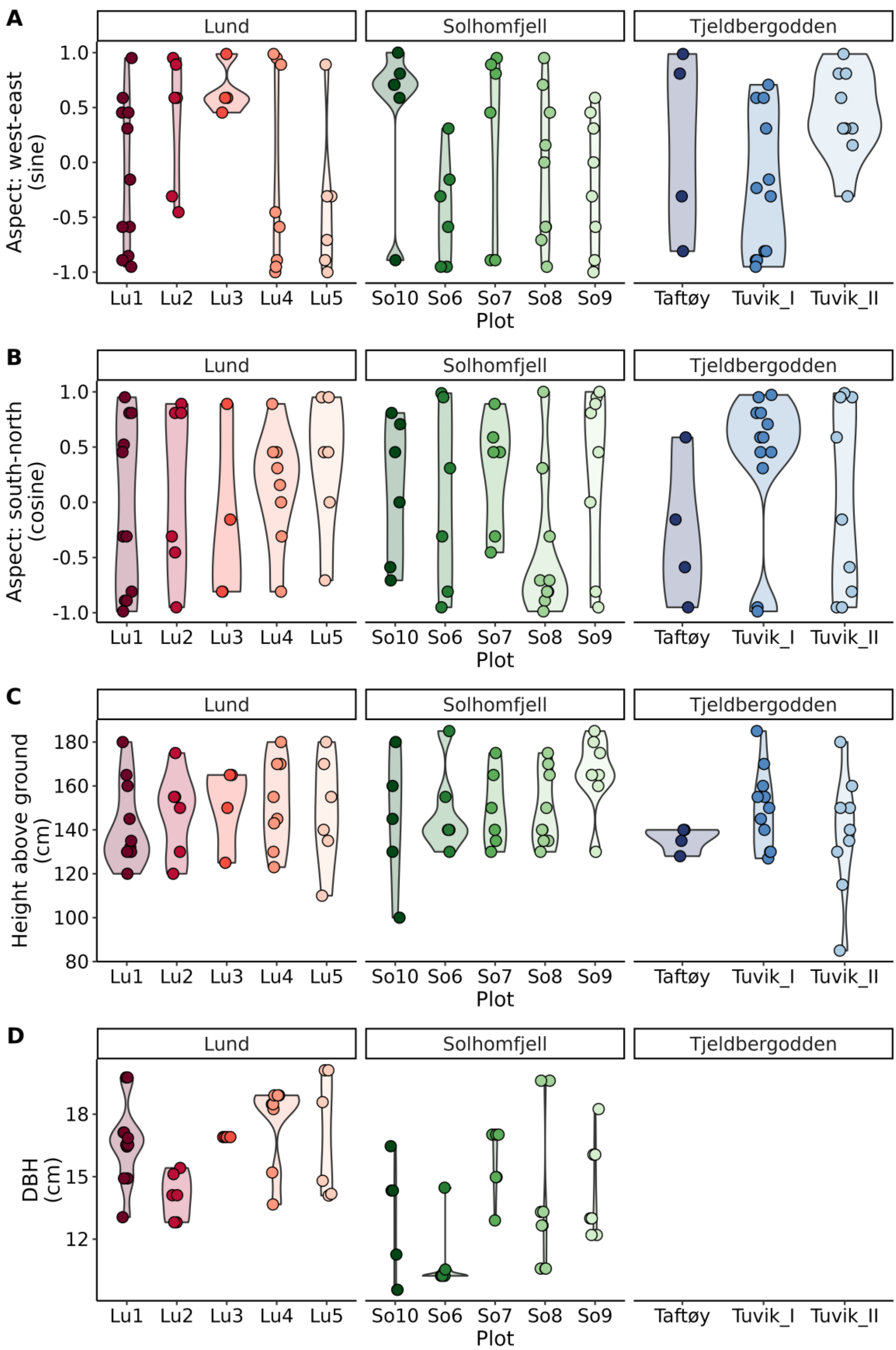

Fig. S3. Violin plots showing variation in measured covariates by site and plot. **A**) West-east aspect (sine-transformed), **B**) South-north aspect (cosine-transformed), **C**) Sampling height above ground, **D)** Diameter at breast height (DBH). DBH was not recorded for Tjeldbergodden.

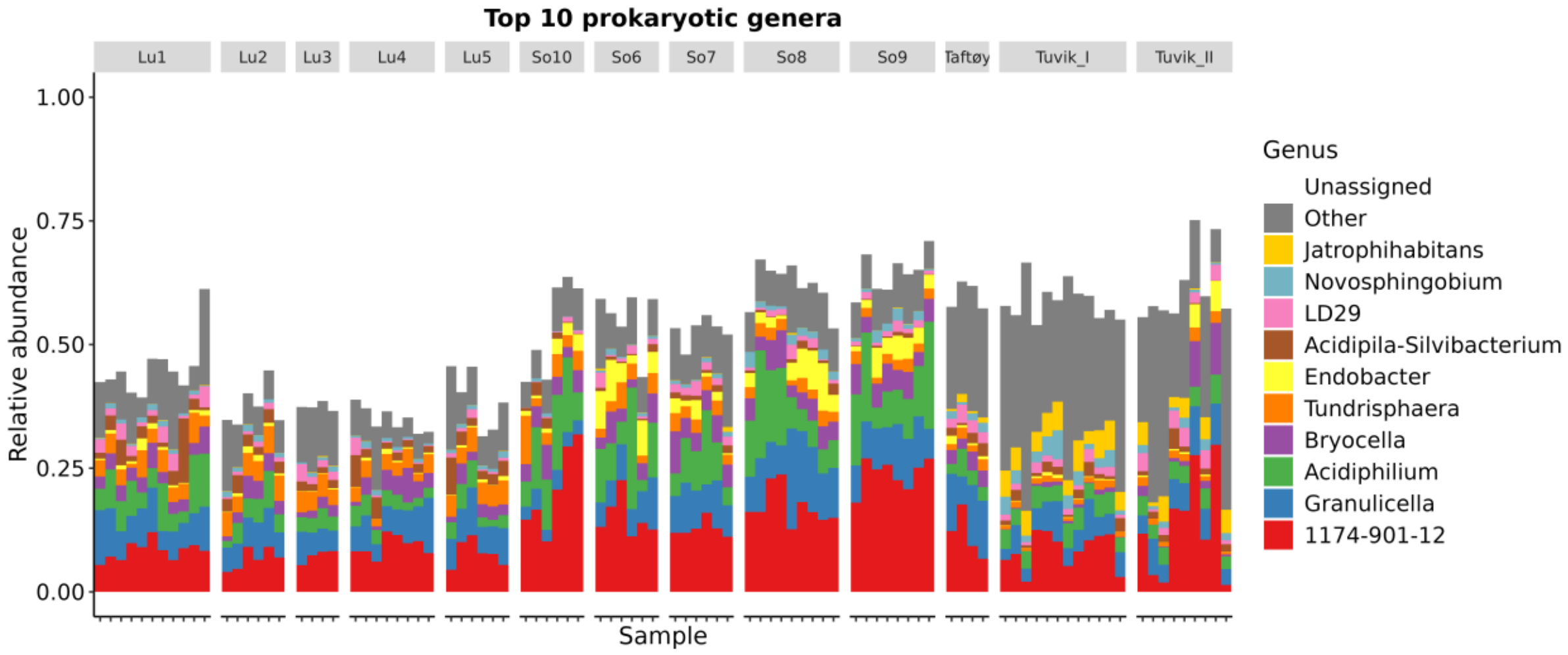

Fig. S4. Relative abundance of the most bacterial abundant genera in each sample. Samples are grouped by plot within each site (Lund: Lu1—5, Solholfjell: So6—10, Tjeldbergodden: Taftøy, Tuvik I and II). The ten most abundant genera are shown, with all other orders grouped into 'other'. ASVs that could not be assigned to a genus are shown in white ('unassigned').

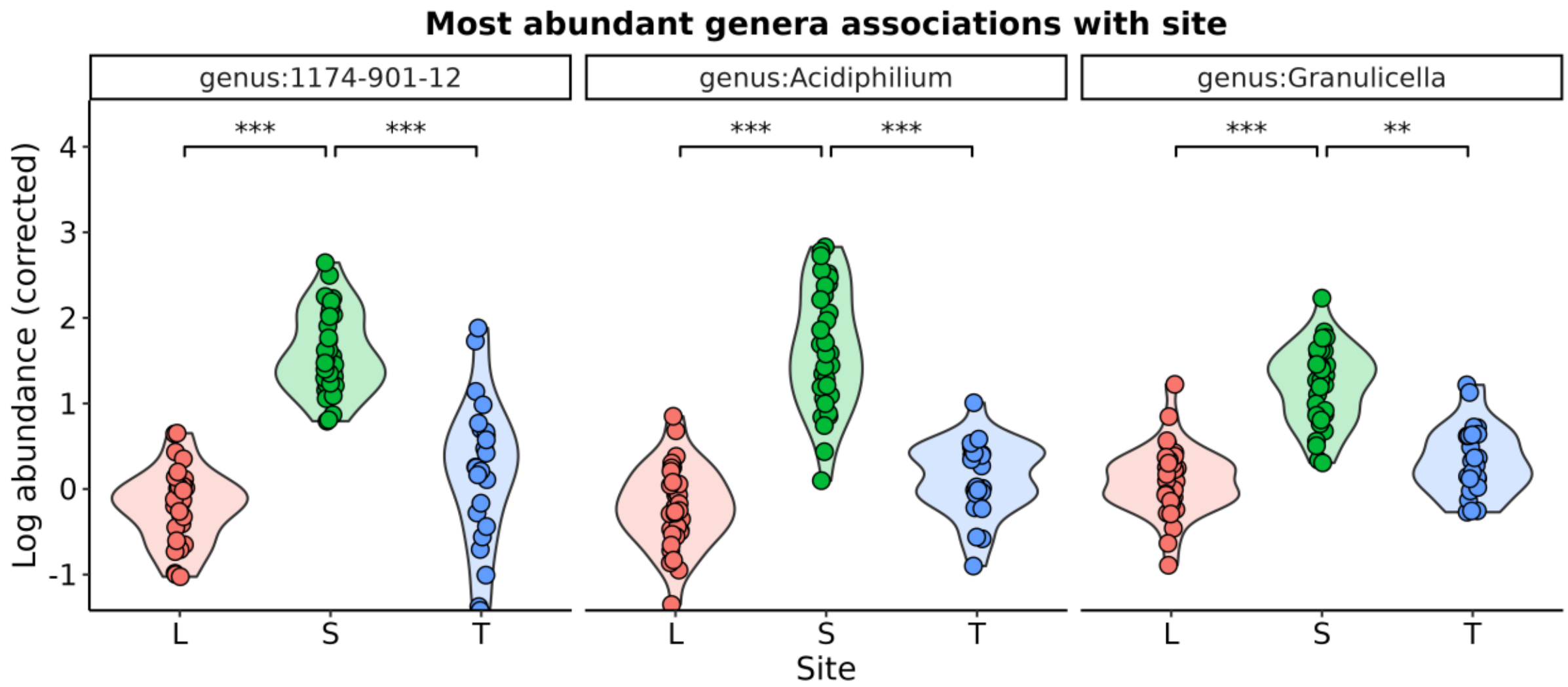

Fig. S5. The three most common bacterial genera associated with site, based on differential abundance analysis with ANCOM. Bacteria abundance has been bias-correlated and log-transformed by ANCOM. Robust statistically significant differences from pairwise comparisons between sites are indicated by brackets (adjusted $p < 0.001$: ***; adjusted $p < 0.01$: **, adjusted $p < 0.05$: *). L: Lund, S: Solhomfjell, T: Tjeldbergodden.

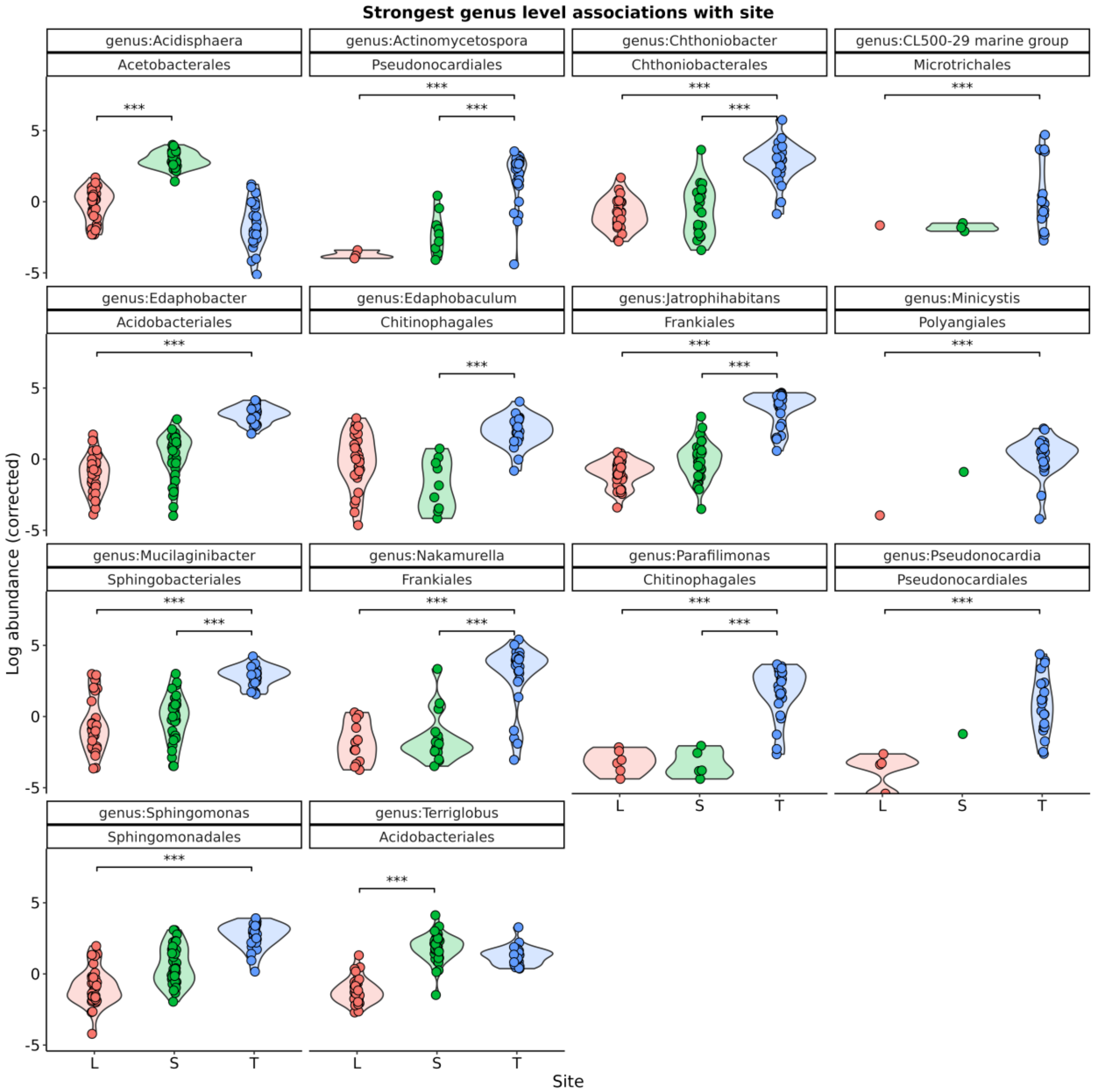

Fig. S6. The 14 bacterial genera most strongly associated with site (> 3-log fold change), based on differential abundance analysis with ANCOM. Bacteria abundance has been bias-correlated and log-transformed by ANCOM. Robust statistically significant differences from pairwise comparisons between sites are indicated by brackets (adjusted $p < 0.001$: ***; adjusted $p < 0.01$: **, adjusted $p < 0.05$: *). L: Lund, S: Solhomfjell, T: Tjeldbergodden. The bacterial order each genus belongs to is shown at the top of each plot.

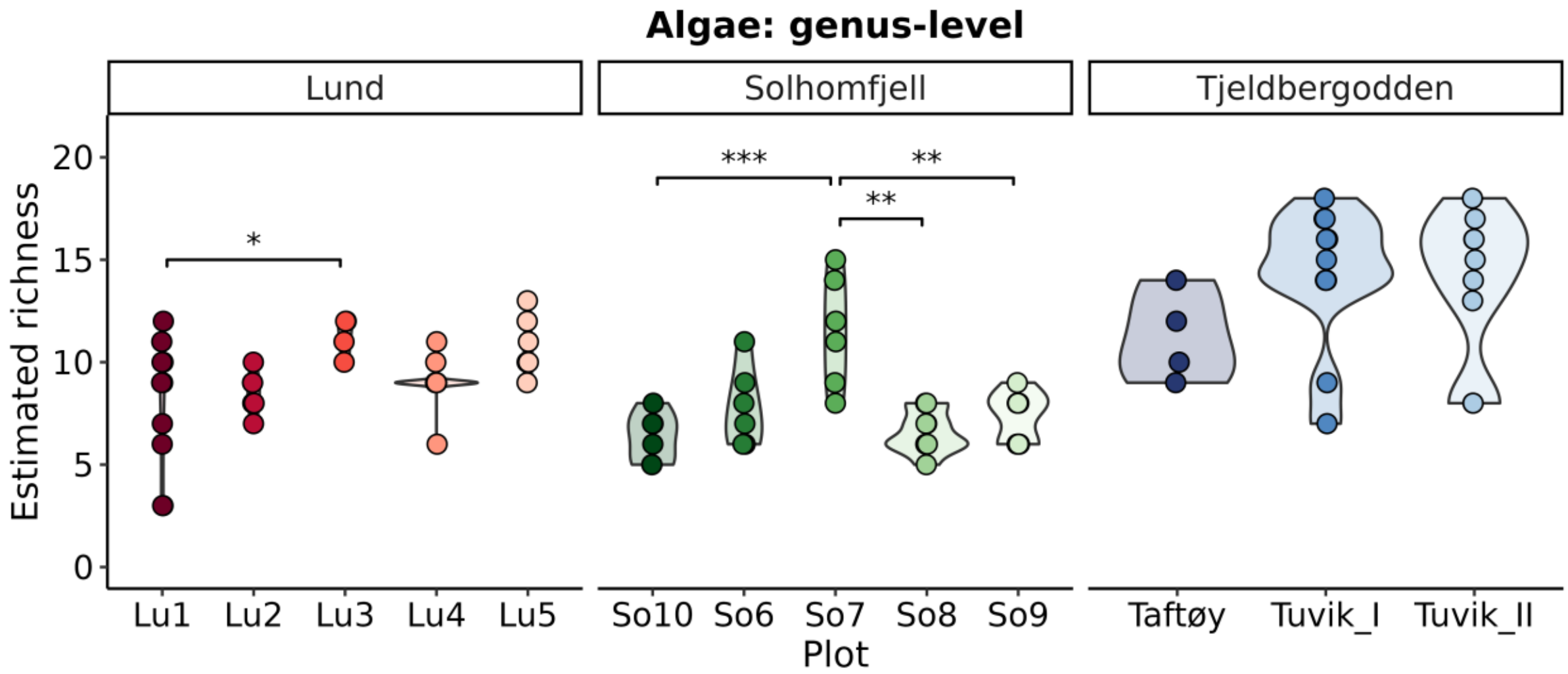

Fig. S7. Violin plots of algae genus-level richness among plots within each site. Statistically significant differences among sites are indicated by brackets ($p < 0.001$: ***; $p < 0.01$: **, $p < 0.05$: *).

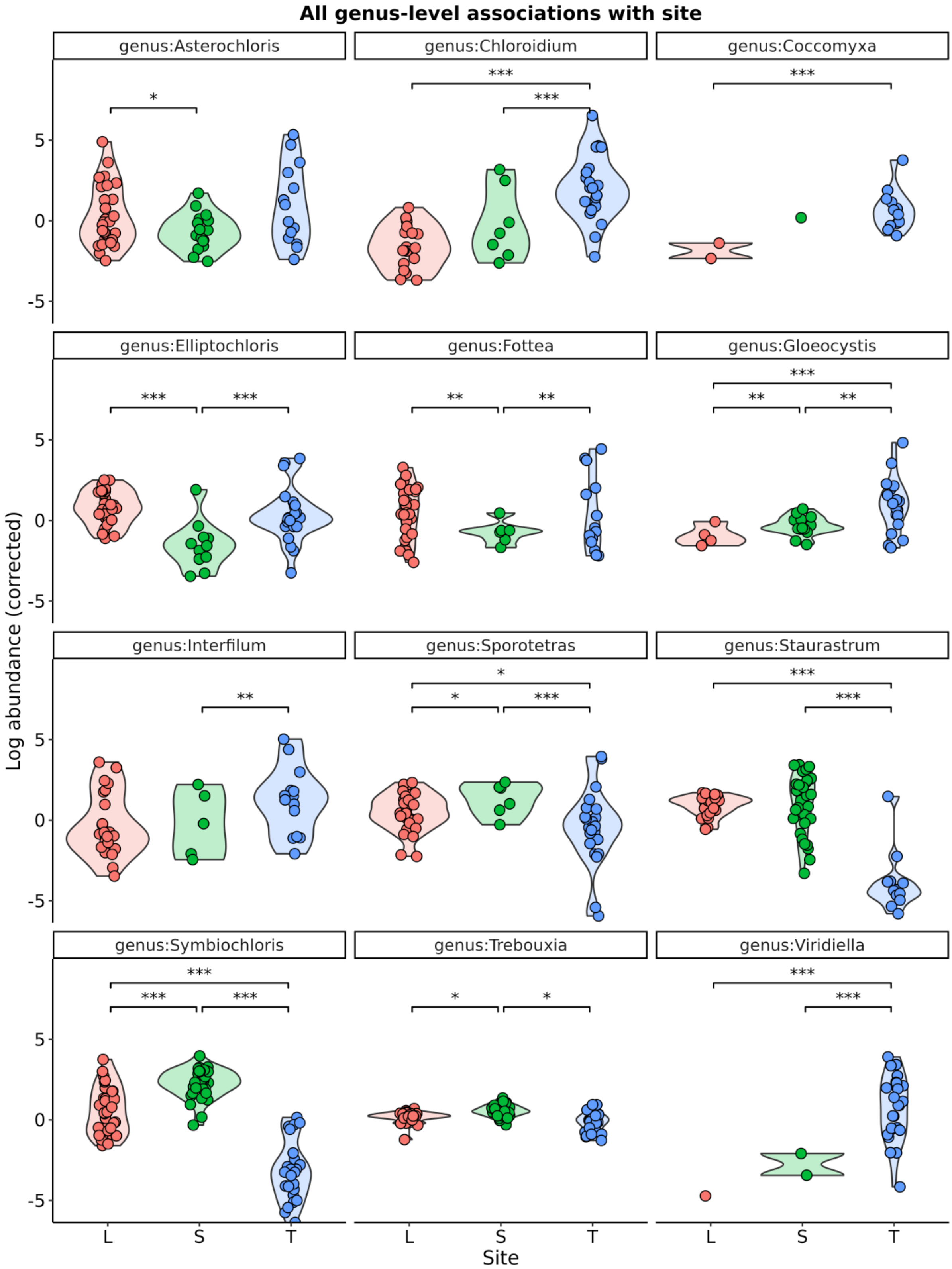

Fig. S8. All 12 green algae genera significantly associated with site, based on differential abundance analysis with ANCOM. Algae abundance has been bias-correlated and log-transformed by ANCOM. Robust statistically significant differences from pairwise comparisons between sites are indicated by brackets (adjusted $p < 0.001$: ***; adjusted $p < 0.01$: **, adjusted $p < 0.05$: *). L: Lund, S: Solhomfjell, T: Tjeldbergodden.

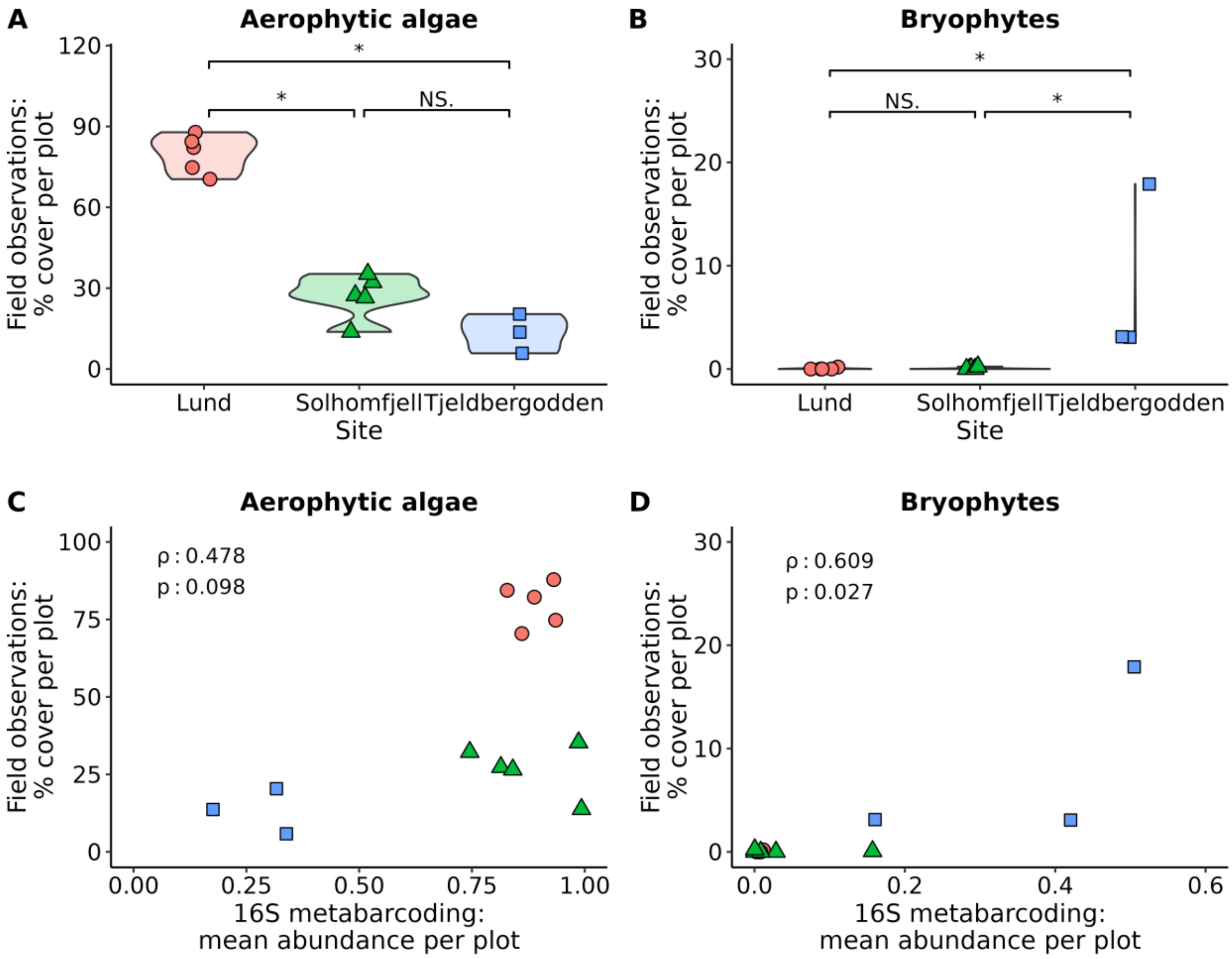

Fig. S9. Observations from the field survey of epiphytes compared to the abundance of photosynthetic eukaryotes detected via 16S metabarcoding. **A-B**) Percent cover per plot of aerophytic green algae (**A**) and bryophytes (mosses, liverworts) (**B**). Statistically significant differences between sites evaluated with the Wilcoxon rank sum test are indicated by brackets ($p < 0.05$: *; NS: not significant, $p > 0.05$). **C-D**) Percent cover per plot from field observations vs mean relative abundance per plot from 16S metabarcoding, for green algae (**C**) and bryophytes (**D**). Spearman correlation (ρ) and p values are shown in the top left corner. Points represent mean values for each plot, coloured and shaped by site (Lund: red circles; Solhomfjell: green triangles; Tjeldbergodden: blue squares).

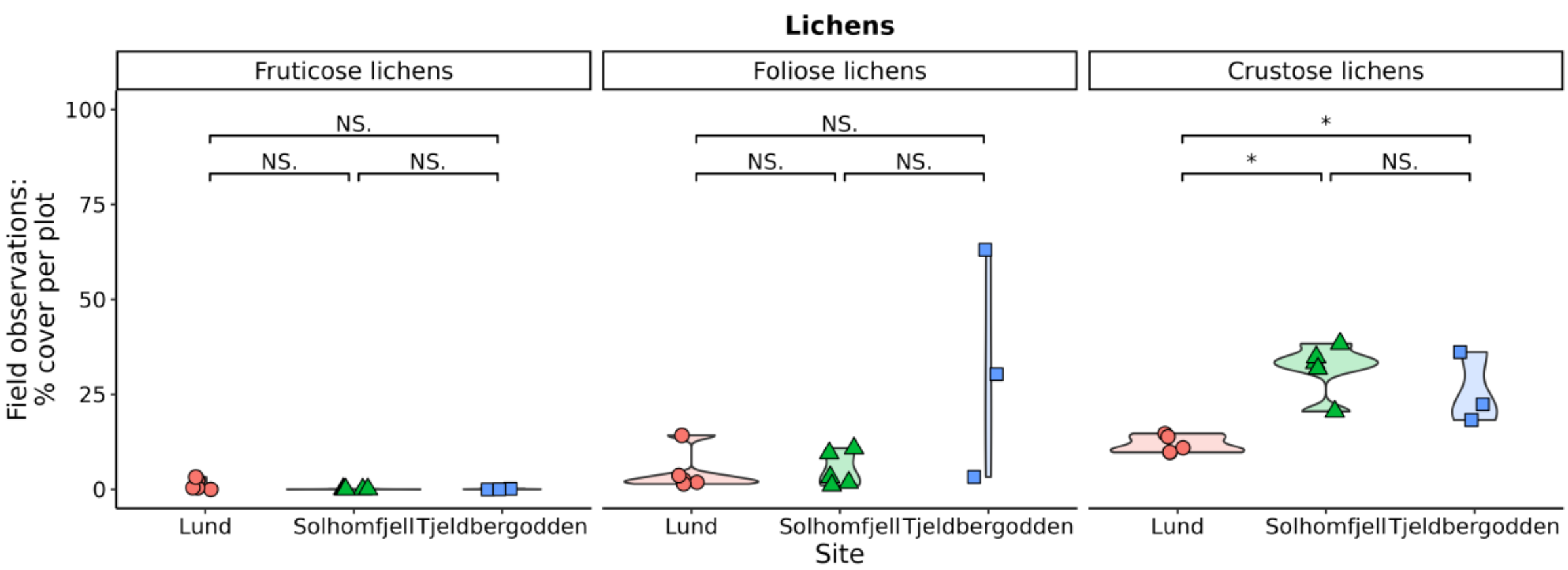

Fig. S10. Observations from the field survey of epiphytic lichens. Percent cover per plot of three lichen groups are shown. Statistically significant differences between sites evaluated with the Wilcoxon rank sum test are indicated by brackets ($p < 0.05$: *; NS: not significant, $p > 0.05$).

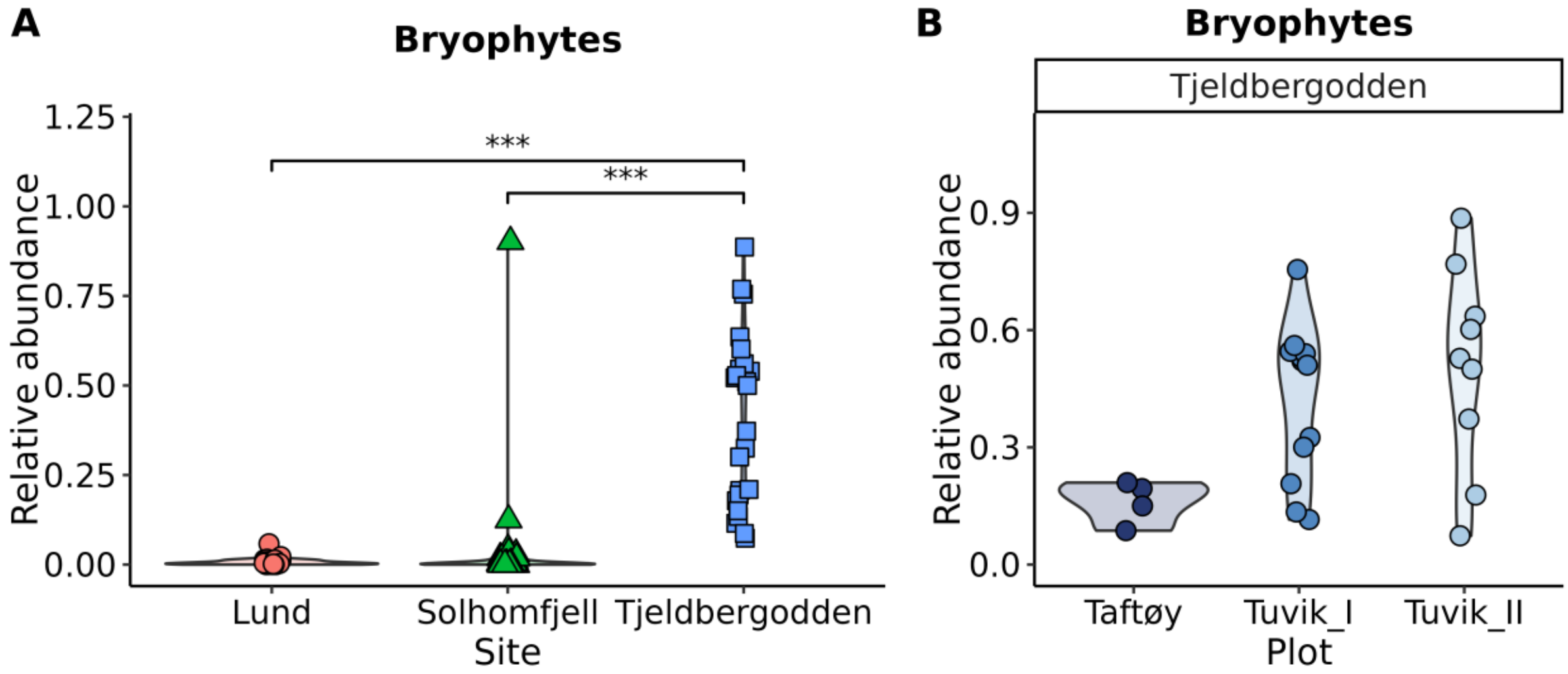

Fig. S11. Proportion of 16S sequences assigned to bryophytes by **A)** site and **B)** plot within Tjeldbergodden. Statistically significant differences among sites are indicated by brackets ($p < 0.001$: ***; $p < 0.01$: **, $p < 0.05$: *). While there was some variation among plots in Tjeldbergodden, the differences were not significant ($p > 0.05$).

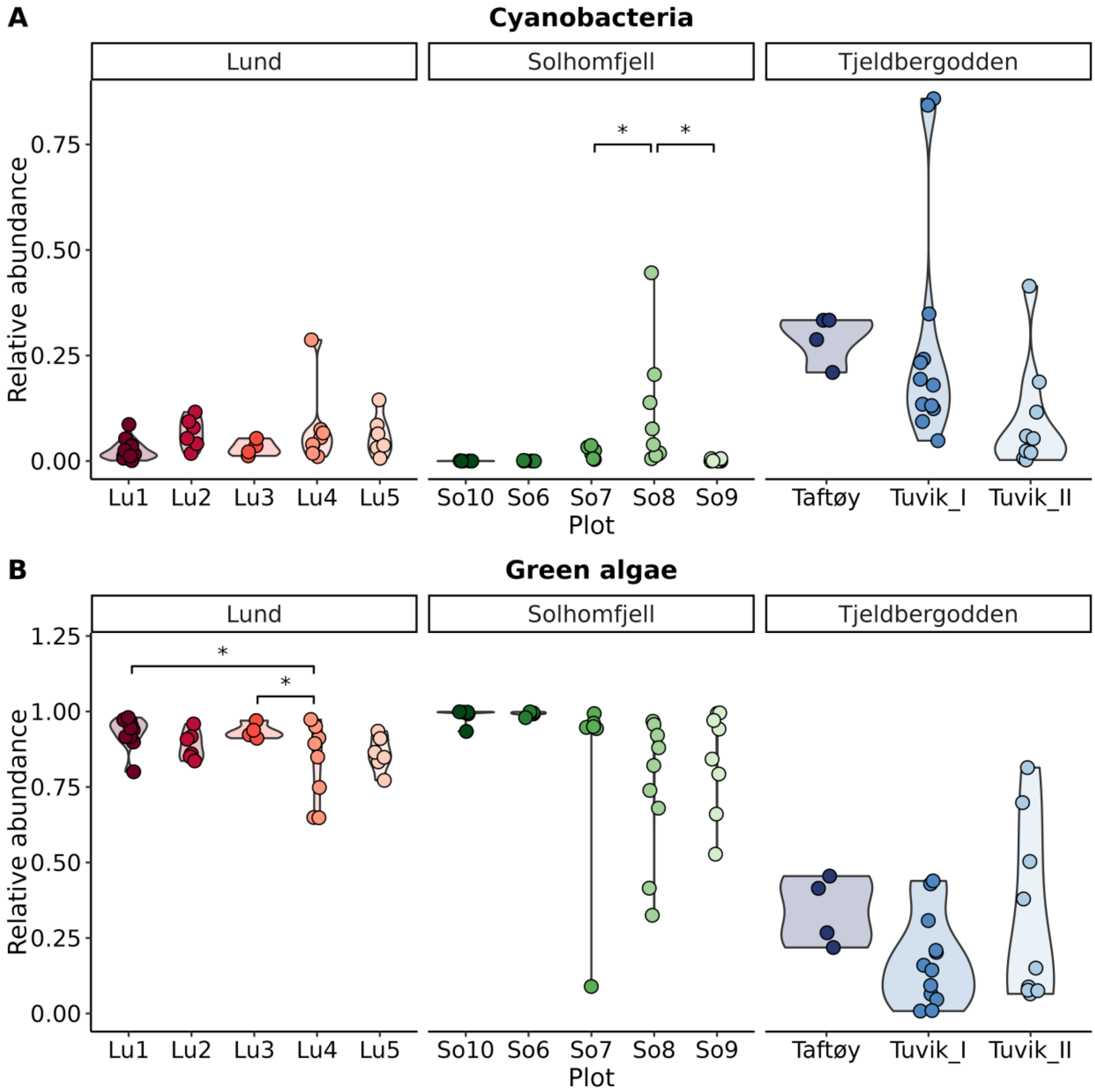

Fig. S12. Proportion of 16S sequences assigned to cyanobacteria (**A**) and green algae (**B**) by plot within each site. Statistically significant differences among plots within each site are indicated by brackets (p < 0.001: ***; p < 0.01: **, p < 0.05: *).

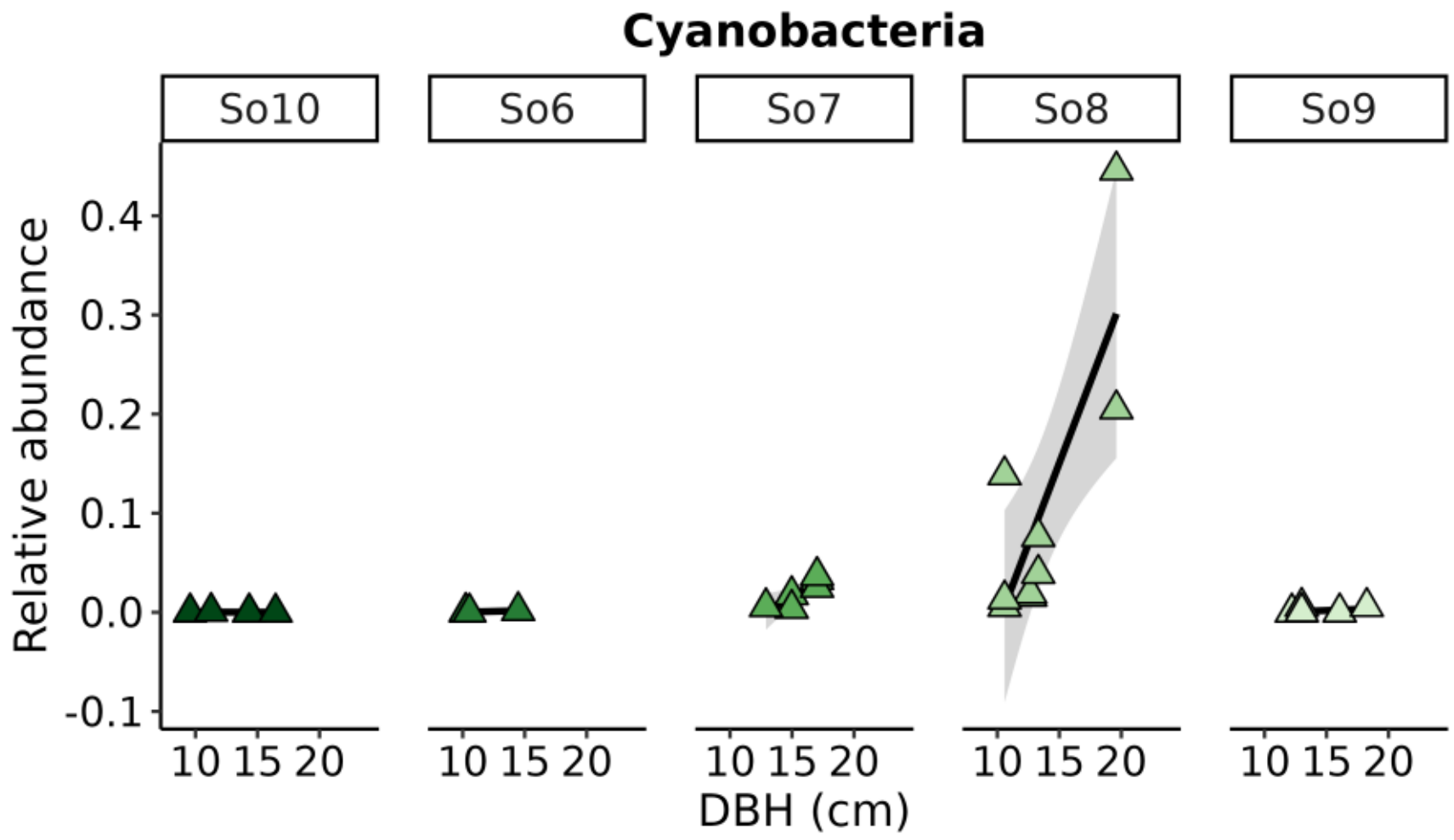

Fig. S13. Proportion of 16S sequences assigned to cyanobacteria by tree size (diameter at breast height, DBH) within each plot in Solhomfjell.

Supplementary tables

Table S1. Characteristics of sampled sites, including climate and vegetation zones, estimated annual S- and N deposition and epiphyte communities.

Site	Köppen-Geiger climate zone 1991-2020 [1]	Estimated total deposition of non sea salt sulfur (mg S / m² / yr) [2]	Estimated total deposition of oxidized + reduced nitrogen (mg N / m² / yr) [2]	Vegetation zone and section [3]	Epiphyte community composition on sampled part of birch trunks [4]
**Lund**	Cold,<br>no dry season,<br>warm summer (Dfb)	1988-1992: > 1400<br>1997-2001: 800 - 900<br>2007-2011: 550 - 650<br>2017-2021: 250 - 350	1988-1992: 2300 - 2400<br>1997-2001: 1600 - 1800<br>2002-2006: 1400 - 1600<br>2017-2021: 850 - 950	Middle boreal zone. Markedly oceanic section (O2)	Epiphyte community strongly dominated by aerophytic algae.
**Solhomfjell**	Cold,<br>no dry season,<br>warm summer (Dfb)	1988-1992: 800 - 900<br>1997-2001: 500 - 600<br>2007-2011: 250 - 350<br>2017-2021: 150 - 250	1988-1992: 1000 - 1200<br>1997-2001: 1000 - 1200<br>2007-2011: 750 - 850<br>2017-2021: 500 - 600	South- / Middle boreal zone. Markedly oceanic section (O2)	Epiphyte community dominated by aerophytic algae and crustose lichens.
**Tjeldbergodden**	Temperate,<br>no dry season,<br>warm summer (Cfb)	1988-1992: 200 - 300<br>1997-2001: 100 - 200<br>2007-2011: < 100<br>2017-2021: 50 - 150	1988-1992: 200 - 400<br>1997-2001: 350 - 450<br>2007-2011: 350 - 450<br>2017-2021: 250 - 350	South boreal zone. Humid, highly oceanic section (O3h)	More variation in community composition among plots. At Taftøy dominance of foliose lichens. At Tuvik II more crustose lichens, aerophytic algae and mosses, while few foliose lichens. Mosses present in all plots.

[1] **Beck HE**, McVicar TR, Vergopolan N, Berg A, Lutsko NJ, Dufour A, Zeng Z, Jiang X, van Dijk AJM & Miralles DG (2023). High-resolution (1 km) Köppen Geiger maps for 1901–2099 based on constrained CMIP6 projections. *Sci Data* 10: 724.

[2] **Aas W**, Hjellbrekke A, Hole LR & Tørseth K (2008). Deposition of major inorganic compounds in Norway 2002–2006. NILU OR 72/2008, *Norwegian Institute for Air Research*, Kjeller; **Blake LR**, Aas W, Denby B, Hjellbrekke A, Mu Q, Simpson D, Ytre-Eide M & Fagerli H (2023). Deposition of sulfur and nitrogen in Norway 2017-2021. METreport 03/2023, *Norwegian Meteorological Institute*, Oslo; **Tørseth K** & Pedersen U (1994). Deposition of sulphur and nitrogen components in Norway 1988-1992. NILU OR 16/94, *Norwegian Institute for Air Research*, Kjeller.

[3] **Moen A** (1999). National Atlas of Norway: Vegetation. Norwegian Mapping Authority, Hønefoss.

[4] **Framstad E** (ed.) (2017). Terrestrial Ecosystems Monitoring in 2016: Ground vegetation, epiphytes, small mammals and birds. Summary of results. NINA Report 1376, *Norwegian Institute for Nature Research*; **Framstad E** (ed.) (2021). Terrestrial Ecosystems Monitoring in 2020: Ground vegetation, epiphytes, small mammals and birds. Summary of results. NINA Report 1972, *Norwegian Institute for Nature Research*; **Pedersen B** & Aarrestad PA (eds.) (2012). Program for environmental monitoring Tjeldbergodden. Monitoring of soil, vegetation and epiphytes 2011. NINA Report 861, *Norwegian Institute for Nature Research*.

Table S2. Location, elevation, field layer vegetation and birch stand characteristics of sampled plots [1].

Site	Plot ID	Coordinates (UTM, center of plot)	Elevation (m.a.s.l.)	Field layer vegetation	Birch stand
Lund	**Lu1**	32V 0350517 6492711	370	Field layer with dominance of the dwarf shrub *Vaccinium myrtillus*.	Mature birch (*Betula pubescence*) forest
	**Lu2**	32V 0350482 6492655	390	Field layer with dominance of *Vaccinium myrtillus*.	Mature birch (*Betula pubescence*) forest
	**Lu3**	32V 0350450 6492588	410	Field layer with co-dominance of *Vaccinium myrtillus* and the fern *Pteridium aquilinum*.	Mature birch (*Betula pubescence*) forest
	**Lu4**	32V 0350585 6492530	390	Field layer with dominance of *Vaccinium myrtillus*.	Mature birch (*Betula pubescence*) forest
	**Lu5**	32V 0350325 6492634	410	Field layer with co-dominance of *Vaccinium myrtillus* and *Pteridium aquilinum*.	Mature birch (*Betula pubescence*) forest
Solhom-fjell	**So6**	32V 0491582 6535841	259	Partly fen vegetation dominated by graminoids and the shrub *Myrica gale*, partly heath vegetation dominated by dwarf shrubs (mostly *Calluna vulgaris* and *Vaccinium myrtillus*) and shrubs (*Juniperus communis*).	Birch (*Betula pendula*) tree stand in transition zone between pine (*Pinus sylvestris*) forest and fen.
	**So7**	32V 0491093 6535144	361	Field layer mostly dominated by *Vaccinium myrtillus*.	Birch (*Betula pendula*) tree stand in mixed pine and spruce (*Picea abies*) forest surrounding a small fen.
	**So8**	32V 0490117 6534837	402	Field layer with dominance of *Vaccinium myrtillus*.	Birch (*Betula pendula*) tree stand located in a depression surrounded by mainly pine forest.
	**So9**	32V 0489468 6534607	399	Field layer with a mixed dominance of *Vaccinium myrtillus*, *Pteridium aquilinum*, and the grass *Molinia caerulea*.	Birch (*Betula pendula*) tree stand in transition zone between a mixed aspen- (*Populus tremula*), pine-, and spruce forest and fen.
	**So10**	32V 0488937 6535205	453	Field layer with a mixed dominance of *Vaccinium myrtillus* and *Molinia caerulea*.	Birch (*Betula pendula*) tree stand in transition zone between pine forest and small fen.
Tjeldber-godden	**Taftøy**	32V 0490697 7032840	85	Field layer dominated by *Vaccinium myrtillus*.	Mature birch (*Betula pubescence*) forest
	**Tuvik I**	32V 0486988 7032356	10	Field layer dominated by *Vaccinium myrtillus*.	Birch (*Betula pubescence*) tree stand surrounded by coastal heath vegetation.
	**Tuvik II**	32V 0487173 7032575	20	Field layer dominated by *Vaccinium myrtillus*.	Birch (*Betula pubescence*) tree stand surrounded by coastal heath vegetation.

[1] **Evju M**, Myklebost HE & Bruteig IE (2015). Monitoring of epiphytes in programme for terrestrial ecosystem monitoring (TOV). Revision of the monitoring protocol 2015. NINA Report 1153, *Norwegian Institute for Nature Research*; **Hilmo O** & Wang R (1992). Terrestrisk naturovervåking. Lavkartlegging i Åmotsdalen og Lund 1991. DN-notat 3-1992, *Direktoratet for naturforvaltning*; **Pedersen B** & Aarrestad PA (eds.) (2012). Program for environmental monitoring Tjeldbergodden. Monitoring of soil, vegetation and epiphytes 2011. NINA Report 861, *Norwegian Institute for Nature Research*. Abbreviations: UTM: Universal Transverse Mercator coordinate system; m.a.s.l.: meters above sea level.

Table S3. Results from linear models of bacterial alpha diversity (genus-level richness).

Covariates	Estimate	Std error	Test statistic	P value
**All sites**				
(Intercept)	58.62	17.29	3.391	0.001
Aspect: west-east (sine)	0.35	3.25	0.109	0.913
Aspect: south-north (cosine)	2.44	3.22	0.756	0.452
Height above ground	0.08	0.11	0.719	0.474
Lund vs Solhomfjell	1.38	5.29	0.260	0.963
**Lund vs Tjeldbergodden**	58.69	5.79	10.140	**< 0.001**
**Tjeldbergodden vs Solhomfjell**	-57.31	5.90	-9.719	**< 0.001**
**Lund**				
(Intercept)	36.60	22.74	1.610	0.120
Aspect: west-east (sine)	5.75	3.07	1.875	0.072
Aspect: south-north (cosine)	-1.87	3.32	-0.565	0.577
Height above ground	0.21	0.12	1.819	0.080
DBH	0.72	1.09	0.661	0.515
Plot (all pairwise comparisons)	NA	NA	NA	> 0.05
**Solhomfjell**				
(Intercept)	27.34	41.77	0.654	0.519
Aspect: west-east (sine)	5.17	5.98	0.865	0.395
Aspect: south-north (cosine)	-7.47	5.56	-1.343	0.191
Height above ground	0.09	0.20	0.466	0.645
DBH	1.64	1.58	1.041	0.307
Plot (all pairwise comparisons)	NA	NA	NA	> 0.05
**Tjeldbergodden**				
(Intercept)	113.91	44.23	2.575	0.019
Aspect: west-east (sine)	-2.72	10.51	-0.259	0.798
Aspect: south-north (cosine)	13.08	8.16	1.604	0.125
Height above ground	-0.05	0.31	-0.165	0.871
Plot (all pairwise comparisons)	NA	NA	NA	> 0.05

Table S4. Results from PERMANOVAs on bacteria and green algae beta diversity at the genus- and ASV-levels within each site, and using site instead of plot for the models with all sites. Variables are presented in the same order that they were included in the PERMANOVA.

Variable	Degrees of freedom	Sum of squares	R^2	F statistic	P value
**Bacteria: genus-level – All sites**					
**Aspect: south-north (cosine)**	1	618	0.019	2.23	**0.023**
Site	2	6776	0.206	12.23	0.263
Aspect: west-east (sine)	1	272	0.008	0.98	0.382
Height above ground	1	258	0.008	0.93	0.664
Interaction: aspect (cosine) * site	2	905	0.027	1.63	0.295
Interaction: aspect (sine) * site	2	575	0.017	1.04	0.884
Residual	85	23553	0.715	NA	NA
Total	94	32957	1.000	NA	NA
**Bacteria: genus-level – Lund**					
**Plot**	4	1117	0.179	1.70	**0.007**
**Aspect: south-north (cosine)**	1	237	0.038	1.44	**0.003**
**Aspect: west-east (sine)**	1	226	0.036	1.37	**0.020**
**DBH**	1	179	0.029	1.09	**0.006**
Height above ground	1	151	0.024	0.92	0.205
Interaction: aspect (cosine) * plot	4	695	0.112	1.06	0.087
Interaction: aspect (sine) * plot	4	655	0.105	0.99	0.197
Residual	18	2963	0.476	NA	NA
Total	34	6221	1.000	NA	NA
**Bacteria: genus-level – Solhomfjell**					
Plot	4	1513	0.179	1.62	0.908
DBH	1	330	0.039	1.42	0.908
Aspect: south-north (cosine)	1	217	0.026	0.93	0.752
Aspect: west-east (sine)	1	191	0.023	0.82	0.798
Height above ground	1	188	0.022	0.81	0.750
Interaction: aspect (cosine) * plot	4	859	0.101	0.92	0.773
Interaction: aspect (sine) * plot	4	973	0.115	1.04	0.696
Residual	18	4199	0.496	NA	NA
Total	34	8470	1.000	NA	NA
**Bacteria: genus-level – Tjeldbergodden**					
Plot	2	1337	0.117	1.45	0.205
Aspect: south-north (cosine)	1	809	0.071	1.76	0.100
Height above ground	1	472	0.041	1.03	0.216
Aspect: west-east (sine)	1	367	0.032	0.80	0.888
Interaction: aspect (cosine) * plot	2	737	0.065	0.80	0.301
Interaction: aspect (sine) * plot	2	788	0.069	0.86	0.408
Residual	15	6896	0.605	NA	NA
Total	24	11406	1.000	NA	NA

Variable	Degrees of freedom	Sum of squares	R^2	F statistic	P value
**Bacteria: ASV-level – All sites**					
**Aspect: south-north (cosine)**	1	3701	0.016	1.86	**0.001**
**Site**	2	49585	0.208	12.46	**0.003**
Aspect: west-east (sine)	1	2219	0.009	1.12	0.203
Height above ground	1	2022	0.008	1.02	0.140
**Interaction: aspect (cosine) * site**	2	6266	0.026	1.57	**0.001**
Interaction: aspect (sine) * site	2	5236	0.022	1.32	0.310
Residual	85	169109	0.710	NA	NA
Total	94	238137	1.000	NA	NA
**Bacteria: ASV-level – Lund**					
**Plot**	4	8367	0.187	1.80	**0.014**
**Aspect: south-north (cosine)**	1	1957	0.044	1.69	**0.001**
Aspect: west-east (sine)	1	1652	0.037	1.42	0.069
**DBH**	1	1281	0.029	1.10	**0.024**
Height above ground	1	1195	0.027	1.03	0.348
Interaction: aspect (cosine) * plot	4	4775	0.107	1.03	0.137
Interaction: aspect (sine) * plot	4	4629	0.103	1.00	0.276
Residual	18	20897	0.467	NA	NA
Total	34	44754	1.000	NA	NA
**Bacteria: ASV-level – Solhomfjell**					
Plot	4	14380	0.235	2.38	0.088
Aspect: south-north (cosine)	1	1703	0.028	1.13	0.080
Aspect: west-east (sine)	1	1674	0.027	1.11	0.482
**DBH**	1	2478	0.040	1.64	**0.027**
Height above ground	1	1252	0.020	0.83	0.756
Interaction: aspect (cosine) * plot	4	6088	0.099	1.01	0.082
Interaction: aspect (sine) * plot	4	6568	0.107	1.09	0.252
Residual	18	27160	0.443	NA	NA
Total	34	61302	1.000	NA	NA
**Bacteria: ASV-level – Tjeldbergodden**					
**Plot**	2	10578	0.129	1.62	**0.002**
**Aspect: south-north (cosine)**	1	4943	0.060	1.52	**0.003**
**Height above ground**	1	3552	0.043	1.09	**0.009**
Aspect: west-east (sine)	1	2943	0.036	0.90	0.868
Interaction: aspect (cosine) * plot	2	5743	0.070	0.88	0.117
Interaction: aspect (sine) * plot	2	5405	0.066	0.83	0.576
Residual	15	48871	0.596	NA	NA
Total	24	82035	1.000	NA	NA

Variable	Degrees of freedom	Sum of squares	R^2	F statistic	P value
**Green algae: genus-level – All sites**					
Aspect: south-north (cosine)	1	69	0.016	1.99	0.064
**Site**	2	964	0.227	13.83	**0.008**
Aspect: west-east (sine)	1	42	0.010	1.20	0.153
Height above ground	1	37	0.009	1.05	0.309
Interaction: aspect (cosine) * site	2	93	0.022	1.33	0.157
Interaction: aspect (sine) * site	2	78	0.018	1.12	0.138
Residual	85	2965	0.698	NA	NA
Total	94	4248	1.000	NA	NA
**Green algae: genus-level – Lund**					
Plot	4	245	0.236	2.28	0.657
Height above ground	1	36	0.035	1.34	0.089
Aspect: south-north (cosine)	1	34	0.032	1.25	0.384
Aspect: west-east (sine)	1	26	0.025	0.97	0.695
DBH	1	22	0.021	0.83	0.329
Interaction: aspect (cosine) * plot	4	70	0.068	0.65	0.952
Interaction: aspect (sine) * plot	4	120	0.116	1.12	0.529
Residual	18	483	0.466	NA	NA
Total	34	1036	1.000	NA	NA
**Green algae: genus-level – Solhomfjell**					
Plot	4	312	0.270	2.70	0.742
Aspect: south-north (cosine)	1	58	0.050	2.00	0.070
Aspect: west-east (sine)	1	27	0.024	0.95	0.135
DBH	1	33	0.029	1.14	0.982
Height above ground	1	20	0.017	0.70	0.470
Interaction: aspect (cosine) * plot	4	74	0.064	0.64	0.970
Interaction: aspect (sine) * plot	4	114	0.099	0.99	0.722
Residual	18	519	0.449	NA	NA
Total	34	1157	1.000	NA	NA
**Green algae: genus-level – Tjeldbergodden**					
Plot	2	105	0.096	1.15	0.101
Aspect: south-north (cosine)	1	68	0.062	1.49	0.079
Aspect: west-east (sine)	1	63	0.058	1.39	0.082
Height above ground	1	38	0.034	0.83	0.557
Interaction: aspect (cosine) * plot	2	67	0.061	0.74	0.326
Interaction: aspect (sine) * plot	2	68	0.063	0.75	0.433
Residual	15	682	0.625	NA	NA
Total	24	1091	1.000	NA	NA

Variable	Degrees of freedom	Sum of squares	R^2	F statistic	P value
**Green algae: ASV-level – All sites**					
**Aspect: south-north (cosine)**	1	301	0.015	1.78	**0.001**
**Site**	2	4179	0.209	12.38	**0.002**
Aspect: west-east (sine)	1	199	0.010	1.18	0.148
Height above ground	1	161	0.008	0.96	0.917
**Interaction: aspect (cosine) * site**	2	476	0.024	1.41	**0.002**
**Interaction: aspect (sine) * site**	2	381	0.019	1.13	**0.040**
Residual	85	14344	0.716	NA	NA
Total	94	20041	1.000	NA	NA
**Green algae: ASV-level – Lund**					
**Plot**	4	1044	0.214	2.16	**0.014**
**Aspect: south-north (cosine)**	1	157	0.032	1.30	**0.001**
Aspect: west-east (sine)	1	144	0.030	1.20	0.109
**DBH**	1	148	0.030	1.22	**0.011**
Height above ground	1	137	0.028	1.14	0.368
Interaction: aspect (cosine) * plot	4	498	0.102	1.03	0.249
Interaction: aspect (sine) * plot	4	580	0.119	1.20	0.116
Residual	18	2176	0.446	NA	NA
Total	34	4883	1.000	NA	NA
**Green algae: ASV-level – Solhomfjell**					
Plot	4	1142	0.203	1.94	0.185
**Aspect: south-north (cosine)**	1	197	0.035	1.34	**0.035**
DBH	1	170	0.030	1.16	0.328
**Aspect: west-east (sine)**	1	161	0.029	1.09	**0.026**
Height above ground	1	153	0.027	1.04	0.293
Interaction: aspect (cosine) * plot	4	586	0.104	1.00	0.246
Interaction: aspect (sine) * plot	4	578	0.103	0.98	0.890
Residual	18	2648	0.470	NA	NA
Total	34	5634	1.000	NA	NA
**Green algae: ASV-level – Tjeldbergodden**					
Plot	2	617	0.116	1.51	0.056
**Aspect: south-north (cosine)**	1	413	0.078	2.02	**0.017**
Aspect: west-east (sine)	1	214	0.040	1.04	0.175
Height above ground	1	193	0.036	0.95	0.882
Interaction: aspect (cosine) * plot	2	405	0.076	0.99	0.112
Interaction: aspect (sine) * plot	2	412	0.077	1.01	0.328
Residual	15	3070	0.577	NA	NA
Total	24	5325	1.000	NA	NA

Table S5. Bacteria genus-level differential abundance results from ANCOM pairwise comparisons of site. L: Lund, S: Solhomfjell, T: Tjeldbergodden. Only genera with robust significant differences (q < 0.05, passed sensitivity analysis) in at least one comparison are shown. The log-fold change (LFC), standard error (SE), test statistic (W), raw p value and adjusted p value (q value) are provided. Comparisons which failed the sensitivity analysis were not robust to the pseudo-count addition and are excluded (NA values).

Genus	Site	Robust	LFC	SE	W	p value	q value	
Abditibacterium	L vs S	Yes	0.421	0.304	1.383	0.173	0.208	
Abditibacterium	L vs T	Yes	2.047	0.378	5.418	1.70E-06	5.50E-06	***
Abditibacterium	T vs S	Yes	1.627	0.421	3.861	3.20E-04	7.10E-04	***
Acidicapsa	L vs S	Yes	2.346	0.314	7.468	4.00E-10	1.30E-09	***
Acidicapsa	L vs T	Yes	0.783	0.359	2.180	0.033	0.04	*
Acidicapsa	T vs S	No	NA	NA	NA	NA	NA	NA
Bryocella	L vs S	Yes	1.428	0.212	6.749	1.50E-09	4.70E-09	***
Bryocella	L vs T	Yes	-0.954	0.324	-2.944	0.004	0.005	**
Bryocella	T vs S	Yes	-2.382	0.359	-6.634	2.50E-09	5.40E-09	***
Edaphobacter	L vs S	Yes	1.381	0.364	3.799	2.70E-04	3.20E-04	***
Edaphobacter	L vs T	Yes	4.238	0.285	14.857	1.80E-25	5.80E-25	***
Edaphobacter	T vs S	Yes	2.857	0.361	7.905	8.20E-12	1.80E-11	***
Granulicella	L vs S	Yes	1.098	0.181	6.085	2.90E-08	9.20E-08	***
Granulicella	L vs T	No	NA	NA	NA	NA	NA	NA
Granulicella	T vs S	Yes	-0.848	0.235	-3.617	4.90E-04	0.001	**
Terriglobus	L vs S	Yes	3.014	0.275	10.953	3.50E-18	1.10E-17	***
Terriglobus	L vs T	Yes	2.489	0.240	10.360	5.80E-17	1.30E-16	***
Terriglobus	T vs S	No	NA	NA	NA	NA	NA	NA
Ca. Solibacter	L vs S	Yes	0.069	0.338	0.205	0.838	1	
Ca. Solibacter	L vs T	Yes	1.028	0.300	3.426	9.30E-04	0.003	**
Ca. Solibacter	T vs S	Yes	0.959	0.386	2.484	0.015	0.033	*
Iamia	L vs S	No	NA	NA	NA	NA	NA	NA
Iamia	L vs T	Yes	1.458	0.298	4.890	4.80E-04	0.001	**
Iamia	T vs S	Yes	-1.546	0.336	-4.599	7.70E-04	0.001	**
CL500-29 marine group	L vs S	No	NA	NA	NA	NA	NA	NA
CL500-29 marine group	L vs T	Yes	3.624	0.395	9.179	2.70E-07	8.50E-07	***
CL500-29 marine group	T vs S	Yes	2.949	0.415	7.103	5.30E-06	1.20E-05	***
Corynebacterium	L vs S	No	NA	NA	NA	NA	NA	NA
Corynebacterium	L vs T	No	NA	NA	NA	NA	NA	NA
Corynebacterium	T vs S	Yes	-1.341	0.271	-4.948	2.70E-04	8.50E-04	***
Mycobacterium	L vs S	Yes	0.244	0.296	0.825	0.411	0.494	
Mycobacterium	L vs T	Yes	2.559	0.372	6.883	9.00E-10	2.90E-09	***
Mycobacterium	T vs S	Yes	2.315	0.428	5.407	5.70E-07	1.20E-06	***
Jatrophihabitans	L vs S	No	NA	NA	NA	NA	NA	NA
Jatrophihabitans	L vs T	Yes	4.563	0.313	14.573	4.60E-25	1.50E-24	***
Jatrophihabitans	T vs S	Yes	3.733	0.388	9.613	2.50E-15	5.50E-15	***
Nakamurella	L vs S	Yes	0.389	0.343	1.136	0.262	0.314	
Nakamurella	L vs T	Yes	4.637	0.492	9.435	1.30E-12	4.30E-12	***
Nakamurella	T vs S	Yes	4.248	0.547	7.765	4.40E-10	9.60E-10	***

Genus	Site	Robust	LFC	SE	W	p value	q value	
Kineosporia	L vs S	Yes	0.265	0.327	0.811	0.422	0.507	
Kineosporia	L vs T	Yes	2.477	0.339	7.308	9.50E-09	3.00E-08	***
Kineosporia	T vs S	Yes	2.212	0.390	5.672	1.60E-06	3.50E-06	***
Amnibacterium	L vs S	No	NA	NA	NA	NA	NA	NA
Amnibacterium	L vs T	Yes	0.861	0.406	2.120	0.041	0.049	*
Amnibacterium	T vs S	Yes	2.150	0.438	4.913	2.00E-05	6.30E-05	***
Actinoplanes	L vs S	No	NA	NA	NA	NA	NA	NA
Actinoplanes	L vs T	Yes	1.424	0.366	3.888	5.90E-04	0.001	**
Actinoplanes	T vs S	No	NA	NA	NA	NA	NA	NA
Marmoricola	L vs S	Yes	0.626	0.287	2.181	0.044	0.053	
Marmoricola	L vs T	Yes	1.657	0.328	5.056	1.20E-04	3.70E-04	***
Marmoricola	T vs S	Yes	1.031	0.361	2.855	0.011	0.025	*
Nocardioides	L vs S	No	NA	NA	NA	NA	NA	NA
Nocardioides	L vs T	Yes	2.415	0.363	6.651	1.10E-06	3.50E-06	***
Nocardioides	T vs S	Yes	1.056	0.416	2.537	0.019	0.023	*
Friedmanniella	L vs S	No	NA	NA	NA	NA	NA	NA
Friedmanniella	L vs T	Yes	1.013	0.296	3.426	0.002	0.007	**
Friedmanniella	T vs S	No	NA	NA	NA	NA	NA	NA
Actinomycetospora	L vs S	Yes	1.176	0.285	4.128	2.70E-04	3.20E-04	***
Actinomycetospora	L vs T	Yes	4.726	0.398	11.869	7.30E-13	2.30E-12	***
Actinomycetospora	T vs S	Yes	3.550	0.453	7.830	9.70E-09	2.10E-08	***
Pseudonocardia	L vs S	No	NA	NA	NA	NA	NA	NA
Pseudonocardia	L vs T	Yes	4.030	0.359	11.225	4.40E-10	9.60E-10	***
Pseudonocardia	T vs S	No	NA	NA	NA	NA	NA	NA
Conexibacter	L vs S	Yes	0.775	0.307	2.520	0.014	0.03	*
Conexibacter	L vs T	Yes	1.550	0.285	5.439	4.70E-07	1.50E-06	***
Conexibacter	T vs S	Yes	0.775	0.337	2.304	0.024	0.03	*
Chthonomonas	L vs S	No	NA	NA	NA	NA	NA	NA
Chthonomonas	L vs T	Yes	0.884	0.503	1.756	0.083	0.099	
Chthonomonas	T vs S	Yes	2.033	0.550	3.697	4.00E-04	0.001	**
Edaphobaculum	L vs S	Yes	-1.523	0.376	-4.046	1.50E-04	1.80E-04	***
Edaphobaculum	L vs T	Yes	2.047	0.359	5.700	3.90E-07	8.50E-07	***
Edaphobaculum	T vs S	Yes	3.570	0.370	9.641	8.40E-14	2.70E-13	***
Parafilimonas	L vs S	Yes	-0.441	0.261	-1.693	0.101	0.121	
Parafilimonas	L vs T	Yes	4.734	0.335	14.128	8.50E-15	2.10E-14	***
Parafilimonas	T vs S	Yes	5.175	0.363	14.271	6.50E-15	2.10E-14	***
Ca. Amoebophilus	L vs S	Yes	-0.181	0.297	-0.609	0.545	0.654	
Ca. Amoebophilus	L vs T	Yes	-0.935	0.259	-3.606	7.20E-04	0.002	**
Ca. Amoebophilus	T vs S	Yes	-0.754	0.303	-2.488	0.016	0.036	*
Cytophaga	L vs S	Yes	-0.401	0.269	-1.493	0.148	0.178	
Cytophaga	L vs T	Yes	-1.172	0.340	-3.448	0.002	0.007	**
Cytophaga	T vs S	No	NA	NA	NA	NA	NA	NA
Hymenobacter	L vs S	No	NA	NA	NA	NA	NA	NA
Hymenobacter	L vs T	Yes	2.110	0.471	4.477	3.20E-05	1.00E-04	***

Genus	Site	Robust	LFC	SE	W	p value	q value	
Hymenobacter	T vs S	Yes	1.806	0.463	3.905	2.30E-04	5.10E-04	***
Flavobacterium	L vs S	No	NA	NA	NA	NA	NA	NA
Flavobacterium	L vs T	Yes	0.762	0.241	3.163	0.008	0.018	*
Flavobacterium	T vs S	No	NA	NA	NA	NA	NA	NA
Mucilaginibacter	L vs S	Yes	0.383	0.437	0.876	0.383	0.46	
Mucilaginibacter	L vs T	Yes	3.467	0.367	9.448	7.50E-15	2.40E-14	***
Mucilaginibacter	T vs S	Yes	3.084	0.370	8.336	1.30E-12	2.80E-12	***
Bdellovibrio	L vs S	Yes	1.345	0.253	5.326	7.50E-07	1.60E-06	***
Bdellovibrio	L vs T	Yes	1.871	0.224	8.344	8.50E-13	2.70E-12	***
Bdellovibrio	T vs S	No	NA	NA	NA	NA	NA	NA
Ktedonobacter	L vs S	Yes	2.877	0.344	8.358	1.60E-05	3.40E-05	***
Ktedonobacter	L vs T	Yes	-1.928	0.265	-7.286	4.60E-05	5.60E-05	***
Ktedonobacter	T vs S	No	NA	NA	NA	NA	NA	NA
Terrisporobacter	L vs S	Yes	-0.365	0.289	-1.265	0.262	0.314	
Terrisporobacter	L vs T	Yes	-2.820	0.202	-13.94	3.40E-05	1.10E-04	***
Terrisporobacter	T vs S	Yes	-2.455	0.241	-10.19	1.60E-04	3.40E-04	***
Gemmatimonas	L vs S	No	NA	NA	NA	NA	NA	NA
Gemmatimonas	L vs T	No	NA	NA	NA	NA	NA	NA
Gemmatimonas	T vs S	Yes	1.276	0.385	3.310	0.004	0.009	**
P3OB-42	L vs S	No	NA	NA	NA	NA	NA	NA
P3OB-42	L vs T	Yes	1.308	0.240	5.458	4.30E-07	1.40E-06	***
P3OB-42	T vs S	Yes	0.826	0.322	2.567	0.012	0.026	*
Haliangium	L vs S	Yes	-0.900	0.320	-2.817	0.006	0.007	**
Haliangium	L vs T	Yes	1.056	0.303	3.486	7.70E-04	0.002	**
Haliangium	T vs S	Yes	1.956	0.355	5.509	3.70E-07	1.20E-06	***
Phaselicystis	L vs S	No	NA	NA	NA	NA	NA	NA
Phaselicystis	L vs T	Yes	1.675	0.404	4.149	1.10E-04	2.50E-04	***
Phaselicystis	T vs S	Yes	2.097	0.412	5.092	4.20E-06	1.30E-05	***
Labilithrix	L vs S	Yes	-0.358	0.275	-1.301	0.207	0.249	
Labilithrix	L vs T	Yes	-1.370	0.271	-5.065	5.10E-05	1.60E-04	***
Labilithrix	T vs S	Yes	-1.012	0.298	-3.401	0.003	0.006	**
Minicystis	L vs S	No	NA	NA	NA	NA	NA	NA
Minicystis	L vs T	Yes	4.336	0.287	15.093	1.20E-11	3.70E-11	***
Minicystis	T vs S	Yes	1.910	0.326	5.865	1.50E-05	1.80E-05	***
Tepidisphaera	L vs S	No	NA	NA	NA	NA	NA	NA
Tepidisphaera	L vs T	Yes	0.720	0.284	2.533	0.018	0.039	*
Tepidisphaera	T vs S	Yes	1.061	0.325	3.264	0.003	0.01	**
Aquisphaera	L vs S	Yes	-0.722	0.292	-2.469	0.016	0.034	*
Aquisphaera	L vs T	Yes	0.207	0.275	0.754	0.453	0.544	
Aquisphaera	T vs S	Yes	0.929	0.354	2.625	0.01	0.033	*
Ca. Nostocoida	L vs S	Yes	-1.421	0.331	-4.287	7.70E-05	2.50E-04	***
Ca. Nostocoida	L vs T	Yes	-1.332	0.402	-3.316	0.002	0.004	**
Ca. Nostocoida	T vs S	No	NA	NA	NA	NA	NA	NA

Genus	Site	Robust	LFC	SE	W	p value	q value	
Singulisphaera	L vs S	No	NA	NA	NA	NA	NA	NA
Singulisphaera	L vs T	Yes	0.790	0.299	2.644	0.011	0.035	*
Singulisphaera	T vs S	No	NA	NA	NA	NA	NA	NA
Tundrisphaera	L vs S	Yes	0.679	0.199	3.405	1.00E-03	0.001	**
Tundrisphaera	L vs T	Yes	-0.735	0.199	-3.685	3.90E-04	8.60E-04	***
Tundrisphaera	T vs S	Yes	-1.414	0.259	-5.466	4.20E-07	1.30E-06	***
Acidicaldus	L vs S	Yes	-1.094	0.314	-3.486	9.30E-04	0.001	**
Acidicaldus	L vs T	Yes	1.814	0.355	5.114	3.60E-06	7.90E-06	***
Acidicaldus	T vs S	Yes	2.908	0.381	7.639	2.20E-10	7.20E-10	***
Acidiphilium	L vs S	Yes	1.852	0.204	9.090	2.40E-14	7.80E-14	***
Acidiphilium	L vs T	No	NA	NA	NA	NA	NA	NA
Acidiphilium	T vs S	Yes	-1.543	0.258	-5.980	4.50E-08	1.00E-07	***
Acidisoma	L vs S	Yes	1.044	0.244	4.285	4.60E-05	1.00E-04	***
Acidisoma	L vs T	Yes	1.364	0.262	5.214	1.20E-06	3.80E-06	***
Acidisoma	T vs S	Yes	0.319	0.322	0.993	0.324	0.388	
Acidisphaera	L vs S	Yes	3.165	0.258	12.26	8.00E-21	1.80E-20	***
Acidisphaera	L vs T	Yes	-1.438	0.347	-4.149	7.60E-05	9.20E-05	***
Acidisphaera	T vs S	Yes	-4.603	0.370	-12.44	3.60E-21	1.10E-20	***
Acidocella	L vs S	Yes	1.570	0.309	5.075	2.20E-06	4.80E-06	***
Acidocella	L vs T	Yes	-1.052	0.316	-3.331	0.001	0.002	**
Acidocella	T vs S	Yes	-2.622	0.392	-6.687	2.10E-09	6.70E-09	***
Endobacter	L vs S	Yes	2.878	0.247	11.633	1.50E-19	4.70E-19	***
Endobacter	L vs T	Yes	0.007	0.316	0.022	0.982	1	
Endobacter	T vs S	Yes	-2.871	0.346	-8.289	1.10E-12	2.40E-12	***
Rhodopila	L vs S	Yes	2.268	0.285	7.959	6.40E-12	2.00E-11	***
Rhodopila	L vs T	Yes	1.236	0.318	3.883	2.00E-04	4.40E-04	***
Rhodopila	T vs S	No	NA	NA	NA	NA	NA	NA
Rhodovastum	L vs S	No	NA	NA	NA	NA	NA	NA
Rhodovastum	L vs T	Yes	1.818	0.283	6.420	6.70E-09	2.10E-08	***
Rhodovastum	T vs S	Yes	0.924	0.368	2.511	0.014	0.017	*
Inquilinus	L vs S	Yes	-1.355	0.331	-4.093	9.70E-05	3.10E-04	***
Inquilinus	L vs T	Yes	-0.790	0.242	-3.264	0.002	0.003	**
Inquilinus	T vs S	Yes	0.565	0.371	1.525	0.131	0.157	
Caedibacter	L vs S	Yes	0.892	0.314	2.840	0.006	0.012	*
Caedibacter	L vs T	Yes	1.296	0.295	4.394	3.10E-05	1.00E-04	***
Caedibacter	T vs S	Yes	0.405	0.368	1.098	0.275	0.33	
Brevundimonas	L vs S	No	NA	NA	NA	NA	NA	NA
Brevundimonas	L vs T	Yes	0.862	0.258	3.338	0.001	0.005	**
Brevundimonas	T vs S	No	NA	NA	NA	NA	NA	NA
Caulobacter	L vs S	No	NA	NA	NA	NA	NA	NA
Caulobacter	L vs T	Yes	1.980	0.272	7.278	5.10E-08	1.60E-07	***
Caulobacter	T vs S	No	NA	NA	NA	NA	NA	NA

Genus	Site	Robust	LFC	SE	W	p value	q value	
Ca. Captivus	L vs S	Yes	0.954	0.272	3.510	0.002	0.006	**
Ca. Captivus	L vs T	No	NA	NA	NA	NA	NA	NA
Ca. Captivus	T vs S	No	NA	NA	NA	NA	NA	NA
1174-901-12	L vs S	Yes	1.789	0.186	9.601	2.10E-15	6.80E-15	***
1174-901-12	L vs T	No	NA	NA	NA	NA	NA	NA
1174-901-12	T vs S	Yes	-1.348	0.276	-4.875	4.70E-06	1.00E-05	***
Methylobacterium[1]	L vs S	Yes	1.447	0.402	3.602	5.30E-04	0.001	**
Methylobacterium[1]	L vs T	Yes	2.382	0.319	7.472	6.80E-11	2.20E-10	***
Methylobacterium[1]	T vs S	Yes	0.935	0.415	2.256	0.027	0.032	*
Methylocella	L vs S	Yes	2.056	0.258	7.981	4.70E-12	1.50E-11	***
Methylocella	L vs T	Yes	0.843	0.245	3.438	8.90E-04	0.001	**
Methylocella	T vs S	Yes	-1.213	0.312	-3.883	2.00E-04	4.40E-04	***
Methylorosula	L vs S	No	NA	NA	NA	NA	NA	NA
Methylorosula	L vs T	No	NA	NA	NA	NA	NA	NA
Methylorosula	T vs S	Yes	-1.133	0.311	-3.638	6.50E-04	0.002	**
Psychroglaciecola	L vs S	No	NA	NA	NA	NA	NA	NA
Psychroglaciecola	L vs T	No	NA	NA	NA	NA	NA	NA
Psychroglaciecola	T vs S	Yes	-0.805	0.315	-2.556	0.016	0.036	*
Roseiarcus	L vs S	Yes	0.230	0.378	0.610	0.543	0.652	
Roseiarcus	L vs T	Yes	1.539	0.323	4.767	7.30E-06	2.30E-05	***
Roseiarcus	T vs S	Yes	1.308	0.471	2.775	0.007	0.015	*
Kaistia	L vs S	No	NA	NA	NA	NA	NA	NA
Kaistia	L vs T	Yes	-1.111	0.318	-3.498	0.002	0.004	**
Kaistia	T vs S	No	NA	NA	NA	NA	NA	NA
Afipia	L vs S	No	NA	NA	NA	NA	NA	NA
Afipia	L vs T	Yes	0.928	0.318	2.917	0.011	0.025	*
Afipia	T vs S	No	NA	NA	NA	NA	NA	NA
Novosphingobium	L vs S	Yes	0.886	0.256	3.456	8.40E-04	0.002	**
Novosphingobium	L vs T	Yes	1.186	0.219	5.424	5.00E-07	1.60E-06	***
Novosphingobium	T vs S	Yes	0.300	0.306	0.980	0.33	0.396	
Polymorphobacter	L vs S	Yes	-1.723	0.349	-4.941	4.10E-06	1.30E-05	***
Polymorphobacter	L vs T	Yes	-0.288	0.300	-0.961	0.339	0.407	
Polymorphobacter	T vs S	Yes	1.434	0.418	3.428	9.60E-04	0.002	**
Sphingoaurantiacus	L vs S	Yes	1.951	0.358	5.450	2.60E-06	8.40E-06	***
Sphingoaurantiacus	L vs T	No	NA	NA	NA	NA	NA	NA
Sphingoaurantiacus	T vs S	No	NA	NA	NA	NA	NA	NA
Sphingomonas	L vs S	Yes	1.443	0.332	4.345	3.70E-05	4.40E-05	***
Sphingomonas	L vs T	Yes	3.680	0.317	11.601	1.70E-19	5.40E-19	***
Sphingomonas	T vs S	Yes	2.237	0.362	6.175	1.90E-08	4.30E-08	***
Verticiella	L vs S	Yes	0.908	0.289	3.144	0.007	0.016	*
Verticiella	L vs T	No	NA	NA	NA	NA	NA	NA
Verticiella	T vs S	Yes	-1.282	0.255	-5.019	1.90E-04	6.00E-04	***
Burkholderia[2]	L vs S	No	NA	NA	NA	NA	NA	NA
Burkholderia[2]	L vs T	Yes	1.580	0.406	3.886	2.00E-04	6.50E-04	***

Genus	Site	Robust	LFC	SE	W	p value	q value	
Burkholderia[2]	T vs S	Yes	0.824	0.428	1.926	0.057	0.096	
Piscinibacter	L vs S	No	NA	NA	NA	NA	NA	NA
Piscinibacter	L vs T	Yes	2.076	0.358	5.799	1.20E-07	3.90E-07	***
Piscinibacter	T vs S	Yes	2.373	0.413	5.740	1.50E-07	3.90E-07	***
Rhizobacter	L vs S	Yes	0.766	0.285	2.687	0.009	0.02	*
Rhizobacter	L vs T	Yes	1.443	0.307	4.702	1.30E-05	4.10E-05	***
Rhizobacter	T vs S	Yes	0.677	0.326	2.079	0.041	0.05	*
Rubrivivax	L vs S	No	NA	NA	NA	NA	NA	NA
Rubrivivax	L vs T	No	NA	NA	NA	NA	NA	NA
Rubrivivax	T vs S	Yes	1.091	0.260	4.195	0.001	0.004	**
Coxiella	L vs S	Yes	-0.115	0.332	-0.346	0.73	0.876	
Coxiella	L vs T	Yes	0.821	0.317	2.588	0.012	0.027	*
Coxiella	T vs S	Yes	0.936	0.345	2.716	0.008	0.027	*
Aquicella	L vs S	Yes	-3.419	0.321	-10.65	1.50E-15	4.80E-15	***
Aquicella	L vs T	Yes	-2.798	0.411	-6.806	5.00E-09	1.10E-08	***
Aquicella	T vs S	No	NA	NA	NA	NA	NA	NA
Acidibacter	L vs S	Yes	0.676	0.282	2.401	0.02	0.043	*
Acidibacter	L vs T	Yes	1.358	0.282	4.813	1.10E-05	3.40E-05	***
Acidibacter	T vs S	No	NA	NA	NA	NA	NA	NA
Ca. Berkiella	L vs S	Yes	-0.986	0.297	-3.322	0.002	0.004	**
Ca. Berkiella	L vs T	No	NA	NA	NA	NA	NA	NA
Ca. Berkiella	T vs S	No	NA	NA	NA	NA	NA	NA
Ca. Ovatusbacter	L vs S	Yes	-0.810	0.292	-2.776	0.008	0.018	*
Ca. Ovatusbacter	L vs T	Yes	0.356	0.379	0.940	0.353	0.423	
Ca. Ovatusbacter	T vs S	Yes	1.165	0.380	3.064	0.004	0.012	*
Legionella	L vs S	No	NA	NA	NA	NA	NA	NA
Legionella	L vs T	Yes	1.014	0.355	2.853	0.005	0.012	*
Legionella	T vs S	Yes	1.408	0.402	3.505	7.40E-04	0.002	**
Pseudomonas	L vs S	Yes	-0.189	0.349	-0.543	0.589	0.707	
Pseudomonas	L vs T	Yes	0.877	0.361	2.430	0.018	0.039	*
Pseudomonas	T vs S	Yes	1.066	0.370	2.880	0.005	0.017	*
Luteibacter	L vs S	No	NA	NA	NA	NA	NA	NA
Luteibacter	L vs T	No	NA	NA	NA	NA	NA	NA
Luteibacter	T vs S	Yes	1.518	0.324	4.680	4.30E-04	9.50E-04	***
Chthoniobacter	L vs S	Yes	0.278	0.351	0.792	0.431	0.518	
Chthoniobacter	L vs T	Yes	3.668	0.343	10.688	5.90E-16	1.90E-15	***
Chthoniobacter	T vs S	Yes	3.390	0.416	8.143	1.60E-11	3.60E-11	***
LD29	L vs S	Yes	0.900	0.252	3.566	5.90E-04	0.001	**
LD29	L vs T	Yes	0.989	0.223	4.434	2.60E-05	8.40E-05	***
LD29	T vs S	Yes	0.090	0.284	0.315	0.753	0.904	

[1] Methylobacterium-Methylorubrum complex
[2] Burkholderia-Caballeronia-Paraburkholderia
Ca. indicates candidus genus.

Table S6. Results from linear models of green algal alpha diversity (genus-level richness).

Covariates	Estimate	Std error	Test statistic	P value
**All sites**				
(Intercept)	9.479	1.876	5.053	< 0.001
Aspect: west-east (sine)	0.076	0.352	0.217	0.829
**Aspect: south-north (cosine)**	1.739	0.347	5.010	**< 0.001**
Height above ground	-0.003	0.012	-0.206	0.838
**Lund vs Solhomfjell**	-1.362	0.571	-2.384	**0.050**
**Lund vs Tjeldbergodden**	4.268	0.626	6.822	**< 0.001**
**Tjeldbergodden vs Solhomfjell**	-5.630	0.633	-8.895	**< 0.001**
**Lund**				
(Intercept)	4.737	3.708	1.278	0.213
Aspect: west-east (sine)	-0.296	0.505	-0.587	0.563
**Aspect: south-north (cosine)**	1.465	0.552	2.654	**0.014**
Height above ground	-0.007	0.019	-0.376	0.710
DBH	0.269	0.178	1.511	0.143
**Plot: Lu1 vs Lu3**	3.586	1.175	3.052	**0.038**
Plot (all other pairwise comparisons)	NA	NA	NA	> 0.05
**Solhomfjell**				
(Intercept)	5.332	3.317	1.608	0.120
Aspect: west-east (sine)	0.309	0.475	0.650	0.521
Aspect: south-north (cosine)	0.551	0.441	1.249	0.223
Height above ground	0.002	0.016	0.138	0.891
DBH	0.037	0.125	0.292	0.772
**Plot: So10 vs So7**	5.049	1.062	4.754	**0.001**
**Plot: So8 vs So7**	4.394	1.009	4.355	**0.002**
**Plot: So9 vs So7**	3.997	0.980	4.080	**0.003**
Plot (all other pairwise comparisons)	NA	NA	NA	> 0.05
**Tjeldbergodden**				
(Intercept)	10.936	3.880	2.819	0.011
Aspect: west-east (sine)	0.386	0.922	0.419	0.680
**Aspect: south-north (cosine)**	3.170	0.715	4.431	**< 0.001**
Height above ground	0.008	0.027	0.310	0.760
Plot (all pairwise comparisons)	NA	NA	NA	> 0.05

Table S7. Algae genus-level differential abundance results from ANCOM pairwise comparisons of site. L: Lund, S: Solhomfjell, T: Tjeldbergodden. Only genera with robust significant differences ($q < 0.05$, passed sensitivity analysis) in at least one comparison are shown. The log-fold change (LFC), standard error (SE), test statistic (W), raw p value and adjusted p value (q value) are provided. Comparisons which failed the sensitivity analysis were not robust to the pseudo-count addition and are excluded (NA values).

Genus	Site	Robust	LFC	SE	W	p value	q value	
Gloeocystis	L vs S	Yes	0.828	0.273	3.030	0.005	0.01	**
Gloeocystis	L vs T	Yes	1.918	0.323	5.944	1.40E-06	4.30E-06	***
Gloeocystis	T vs S	Yes	1.090	0.364	2.991	0.005	0.01	**
Sporotetras	L vs S	Yes	0.666	0.307	2.169	0.035	0.035	*
Sporotetras	L vs T	Yes	-1.194	0.443	-2.696	0.009	0.019	*
Sporotetras	T vs S	Yes	-1.860	0.449	-4.139	1.20E-04	3.70E-04	***
Asterochloris	L vs S	Yes	-0.935	0.401	-2.335	0.024	0.047	*
Asterochloris	L vs T	No	NA	NA	NA	NA	NA	NA
Asterochloris	T vs S	No	NA	NA	NA	NA	NA	NA
Symbiochloris	L vs S	Yes	1.671	0.337	4.958	3.50E-06	3.50E-06	***
Symbiochloris	L vs T	Yes	-3.772	0.470	-8.027	4.70E-12	9.30E-12	***
Symbiochloris	T vs S	Yes	-5.443	0.464	-11.73	1.60E-19	4.80E-19	***
Trebouxia	L vs S	Yes	0.408	0.177	2.307	0.023	0.047	*
Trebouxia	L vs T	No	NA	NA	NA	NA	NA	NA
Trebouxia	T vs S	Yes	-0.719	0.259	-2.773	0.007	0.02	*
Chloroidium	L vs S	No	NA	NA	NA	NA	NA	NA
Chloroidium	L vs T	Yes	3.326	0.460	7.228	9.10E-09	2.70E-08	***
Chloroidium	T vs S	Yes	1.789	0.491	3.643	7.60E-04	7.60E-04	***
Viridiella	L vs S	No	NA	NA	NA	NA	NA	NA
Viridiella	L vs T	Yes	5.888	0.392	15.018	1.10E-12	3.20E-12	***
Viridiella	T vs S	Yes	4.126	0.422	9.786	2.80E-09	5.70E-09	***
Coccomyxa	L vs S	No	NA	NA	NA	NA	NA	NA
Coccomyxa	L vs T	Yes	1.427	0.251	5.677	2.00E-04	6.10E-04	***
Coccomyxa	T vs S	No	NA	NA	NA	NA	NA	NA
Elliptochloris	L vs S	Yes	-2.152	0.303	-7.111	2.10E-09	6.20E-09	***
Elliptochloris	L vs T	Yes	-0.656	0.368	-1.783	0.08	0.08	
Elliptochloris	T vs S	Yes	1.496	0.398	3.757	4.10E-04	8.10E-04	***
Interfilum	L vs S	No	NA	NA	NA	NA	NA	NA
Interfilum	L vs T	No	NA	NA	NA	NA	NA	NA
Interfilum	T vs S	Yes	1.484	0.394	3.772	5.80E-04	0.002	**
Staurastrum	L vs S	Yes	0.225	0.363	0.620	0.537	0.537	
Staurastrum	L vs T	Yes	-4.781	0.331	-14.43	2.40E-23	7.20E-23	***
Staurastrum	T vs S	Yes	-5.006	0.443	-11.31	7.20E-18	1.40E-17	***
Fottea	L vs S	Yes	-1.424	0.366	-3.891	3.50E-04	0.001	**
Fottea	L vs T	No	NA	NA	NA	NA	NA	NA
Fottea	T vs S	Yes	1.244	0.375	3.317	0.002	0.004	**

Table S8. Results from linear models of relative abundance of bryophytes, cyanobacteria and green algae, based on 16S assignments.

Covariates	Estimate	Std error	Test statistic	P value
**Bryophytes: all sites**				
(Intercept)	-0.0207	0.121	-0.172	0.864
Aspect: west-east (sine)	-0.0088	0.023	-0.390	0.697
Aspect: south-north (cosine)	0.0221	0.022	0.985	0.327
Height above ground	0.0002	0.001	0.225	0.822
Lund vs Solhomfjell	0.0288	0.037	0.779	0.716
**Lund vs Tjeldbergodden**	0.4016	0.040	9.948	**< 0.001**
**Tjeldbergodden vs Solhomfjell**	-0.3728	0.041	-9.065	**<0.001**
**Bryophytes: Tjeldbergodden**				
(Intercept)	0.1588	0.354	0.448	0.659
Aspect: west-east (sine)	0.0227	0.084	0.270	0.790
Aspect: south-north (cosine)	0.0403	0.065	0.618	0.544
Height above ground	0.0001	0.002	0.027	0.979
Plot: Taftøy vs Tuvik I	0.2415	0.143	1.692	0.232
Plot: Taftøy vs Tuvik II	0.3270	0.140	2.336	0.074
Plot: Tuvik II vs Tuvik I	-0.0855	0.116	-0.738	0.743
**Cyanobacteria: all sites**				
**All sites**				
(Intercept)	0.0403	0.101	0.398	0.691
Aspect: west-east (sine)	-0.0130	0.019	-0.684	0.496
Aspect: south-north (cosine)	0.0294	0.019	1.561	0.122
Height above ground	0.0001	0.001	0.088	0.930
Lund vs Solhomfjell	-0.0189	0.031	-0.610	0.815
**Lund vs Tjeldbergodden**	0.1671	0.034	4.932	**< 0.001**
**Tjeldbergodden vs Solhomfjell**	-0.1860	0.035	-5.388	**< 0.001**
**Lund**				
(Intercept)	-0.0538	0.090	-0.600	0.554
Aspect: west-east (sine)	0.0235	0.012	1.942	0.063
**Aspect: south-north (cosine)**	0.0311	0.013	2.379	**0.025**
Height above ground	0.0001	0.000	0.153	0.880
DBH	0.0045	0.004	1.051	0.303
Plot (all pairwise comparisons)	NA	NA	NA	> 0.05
**Solhomfjell**				
(Intercept)	-0.1808	0.126	-1.434	0.164
Aspect: west-east (sine)	-0.0042	0.018	-0.235	0.816
Aspect: south-north (cosine)	0.0009	0.017	0.056	0.956
Height above ground	-0.0001	0.001	-0.233	0.817
**DBH**	0.0162	0.005	3.398	**0.002**
**Plot: So8 vs So7**	-0.1180	0.038	-3.077	**0.035**
**Plot: So9 vs So8**	0.1129	0.036	3.160	**0.029**
Plot (all other pairwise comparisons)	NA	NA	NA	> 0.05

Covariates	Estimate	Std error	Test statistic	P value
**Tjeldbergodden**				
(Intercept)	0.2464	0.336	0.734	0.472
Aspect: west-east (sine)	-0.0064	0.080	-0.080	0.937
Aspect: south-north (cosine)	0.0935	0.062	1.511	0.147
Height above ground	0.0005	0.002	0.228	0.822
Plot (all pairwise comparisons)	NA	NA	NA	> 0.05
**Green algae: all sites**				
**All sites**				
(Intercept)	1.0079	0.139	7.262	1.38E-10
Aspect: west-east (sine)	0.0370	0.026	1.420	0.159
Aspect: south-north (cosine)	-0.0418	0.026	-1.618	0.109
Height above ground	-0.0008	0.001	-0.857	0.394
Lund vs Solhomfjell	-0.0230	0.042	-0.542	0.851
**Lund vs Tjeldbergodden**	-0.6390	0.046	-13.753	**< 0.001**
**Tjeldbergodden vs Solhomfjell**	0.6160	0.047	13.013	**< 0.001**
**Lund**				
(Intercept)	0.7749	0.114	6.778	3.41E-07
**Aspect: west-east (sine)**	-0.0482	0.015	-3.126	**0.004**
**Aspect: south-north (cosine)**	-0.0617	0.017	-3.704	**0.001**
Height above ground	0.0006	0.001	1.018	0.318
DBH	0.0037	0.005	0.676	0.505
**Plot: Lu4 vs Lu1**	-0.1012	0.028	-3.607	**0.010**
**Plot: Lu4 vs Lu3**	0.1258	0.038	3.285	**0.022**
Plot (all other pairwise comparisons)	NA	NA	NA	> 0.05
**Solhomfjell**				
(Intercept)	1.2504	0.392	3.187	0.004
Aspect: west-east (sine)	0.0957	0.056	1.705	0.100
Aspect: south-north (cosine)	-0.0002	0.052	-0.004	0.997
Height above ground	-0.0006	0.002	-0.334	0.741
DBH	-0.0172	0.015	-1.160	0.257
Plot (all pairwise comparisons)	NA	NA	NA	> 0.05
**Tjeldbergodden**				
(Intercept)	0.5009	0.322	1.557	0.136
Aspect: west-east (sine)	0.0116	0.076	0.152	0.880
Aspect: south-north (cosine)	-0.1119	0.059	-1.886	0.075
Height above ground	-0.0014	0.002	-0.647	0.525
Plot (all pairwise comparisons)	NA	NA	NA	> 0.05
